# Association between income, employment status, and asthma outcomes: a systematic review and meta-analysis

**DOI:** 10.1016/j.lanepe.2025.101367

**Published:** 2025-06-26

**Authors:** Zakariah Gassasse, Isaa D. Khan, Elliot Mok, Ayyash M. Asick, Andrew Tan, Aziz Sheikh, Ian Sinha, Gwyneth A. Davies, Hannah Whittaker, Constantinos Kallis, Jennifer K. Quint

**Affiliations:** a Imperial College London, School of Public Health, 90 Wood Lane, London, W12 0BZ, United Kingdom; b Imperial College London, Sir Alexander Fleming Building, South Kensington Campus, London, United Kingdom; c Nuffield Department of Primary Care Health Sciences, University of Oxford, Oxford, United Kingdom; d Faculty of Health and Life Sciences, University of Liverpool, Liverpool, United Kingdom; e Population Data Science, Faculty of Medicine, Health and Life Science, Swansea University, Swansea, United Kingdom

**Keywords:** Asthma, Income, Employment, Exacerbations, Admissions, Mortality, Inequalities, Socioeconomic status, Deprivation, Index of multiple deprivation, Domains, Material disadvantage

## Abstract

**Background:**

Health inequalities are deeply entrenched in society, and finding ways to reduce these, therefore, represents a major health policy challenge. Focusing on the two highest weighted Index of Multiple Deprivation domains, namely income and employment, we sought to synthesise the evidence on the association between these major determinants of socioeconomic status and asthma outcomes.

**Methods:**

In this systematic review and meta-analysis, we searched key concepts related to employment, income, and asthma outcomes using Medline and Embase for studies published between January 1, 2010 and April 3, 2025. Studies were eligible for inclusion if they were in English and described an association between income and/or employment and asthma outcomes, including exacerbations, hospital admissions and mortality, in people with asthma. Risk Of Bias In Non-randomized Studies–of Exposures (ROBINS-E), Risk of Bias (RoB) and adapted RoB tools were used to assess the risk of bias in the included studies. Using the restricted maximum likelihood method, we meta-analysed the rate of exacerbations and explored heterogeneity between age-related population groups: children (under 18 years) and adults (18 years and older). This study was registered with PROSPERO, CRD42024527300.

**Findings:**

We identified 4153 potentially eligible studies, of which 3141 were screened. 30 studies met the inclusion criteria, with most having a low risk of bias. 19 studies reported income as the exposure and exacerbation as the outcome, of which ten were included in the meta-analysis. People in the lowest income group were more likely to experience an asthma exacerbation than those in the highest income group: OR 1.25; 95% CI 1.13–1.37 overall and when stratified by age: children (1.36 [1.23–1.50]) and adults (1.19 [1.05–1.33]). Only three studies investigated the role of unemployment and were narratively synthesised. While unemployment was associated with increased emergency care visits, its role in predicting exacerbations was less clear.

**Interpretation:**

There is a need for upstream interventions aiming to reduce income inequalities and to investigate their impact on reducing asthma inequalities.

**Funding:**

10.13039/501100023699Health Data Research UK, Inflammation and Immunity Driver Programme.

**Research in context**

**Evidence before this study**

Embase and MEDLINE (via the OVID interface) were used to identify previous systematic reviews and meta-analyses published in English from 2010 until 2025 on the association between socioeconomic status and asthma outcomes. The search terms deployed were: ((asthma) AND (mortality OR ∗admissions OR exacerbations OR hospitali?ations) AND (socioeconomic status OR ses) AND (inequalities OR deprivation OR imd OR index of multiple deprivation) AND (systematic review OR meta-analysis)). Two previous studies were identified. One study investigated socioeconomic status as a whole and by specific domains, such as education and employment, and included studies published between 2000 and 2005. The main findings revealed that lower socioeconomic status was associated with more secondary care healthcare utilisation. The other study focussed on asthma prevalence and allergies, with the former being associated with a lower socioeconomic position (an indicator of socioeconomic status) and the latter being associated with a higher socioeconomic position.

**Added value of this study**

This is an up-to-date review of the role of socioeconomic inequalities in asthma outcomes for children and adults from 2010 to 2025. Amid regional and global economic and political challenges, this timely review focuses on the two highest weighted components of the Index of Multiple Deprivation (IMD) domains, income and employment, and synthesises these specific factors contributing to socioeconomic status and the association with asthma outcomes. The association was assessed overall and by children and adults separately, indicating that lower income is associated with a higher risk of asthma exacerbations in children and adults.

**Implications of all the available evidence**

Lower income and unemployment reflect the material disadvantage (the lack of income, goods and services), which can have synergistic effects on poorer asthma outcomes in four ways: 1) the high costs of healthy living; 2) indoor and air outdoor pollution and loss of protective factors; 3) the pathobiology of poverty; and 4) inverse care law. Addressing the material disadvantages is paramount to improving widespread disparities and, as such, requires a multipronged approach. Researchers will need to investigate further the underlying mechanisms at IMD domain level, including the direct role of unemployment and income on hospital admissions and mortality in a heterogeneous adult population. Clinicians should recognise and consider options to mitigate these material disadvantages that manifest in the clinical pathway and impact outcomes. Policymakers should consider policies, such as better housing conditions and unemployment-related stress, targeting the worst deprived groups to improve outcomes and reduce long-term inequalities.

## Introduction

Addressing the impact of deprivation on asthma outcomes has been identified as both a research and health policy priority in the UK.^1^ An estimated 5.4 million people have asthma, approximately 8 in every 100, placing the UK among the countries with the highest asthma prevalence worldwide.^2^ The UK also has one of the highest asthma death rates for 5- to 34-year-olds in Europe.^3^ The inequalities over the last decade due to austerity, poverty and COVID-19 have contributed to over a million premature deaths and are likely to have led to poorer asthma outcomes, notably in management, in the most deprived deciles.^4^, ^5^, ^6^ Asthma costs the UK health service an estimated £1.1 billion annually, with almost three-quarters spent on providing primary care services (60% prescribing and 14% consultations).^7^

England’s National Health Service (NHS) launched the Core20PLUS5 in 2021 as part of its 10-year Long Term Plan.^8^ This initiative aims to tackle healthcare inequalities in the most deprived IMD quintile (Core20) among the most vulnerable (PLUS) based on five key clinical outcomes requiring ‘accelerated improvement’, including chronic respiratory disease.^9^

The role of socioeconomic inequalities in asthma care and outcomes is well-documented in the literature.^10^, ^11^, ^12^ Socioeconomic status (SES), the most common measure of socioeconomic inequalities, is considered a social determinant of health and a risk factor for worse asthma outcomes.^13^ Previous systematic reviews have reviewed the association between SES broadly and asthma prevalence and allergies as well as health care utilisation, exacerbations and mortality.^14^^,^^15^ However, SES is a social construct encompassing many factors.^16^ To that end, SES is most commonly proxied by the Index of Multiple Deprivation (IMD).^17^ IMD, last recorded in 2019, comprises seven unevenly weighted domains, i.e., income (22.5%), employment (22.5%), health (13.5%), education (13.5%), crime (9.3%), barriers to housing and services (9.3%) and living environment (9.3%).^18^ As the interest is on where to improve and what can be modified, it is important to tease out the components of IMD, starting with those most highly weighted, namely income and employment.

No previous systematic reviews have been conducted focused on the association between employment/income domains of socioeconomic status and asthma outcomes. We sought to critically assess and synthesise the evidence on the association between income and employment as the two key contributors to SES and asthma outcomes from 2010 to 2025.

## Methods

This systematic review is reported following the Preferred Reporting Items for Systematic Reviews and Meta-Analyses (PRISMA) 2020 statement and its checklist (Supplementary material Table S1).^19^ The protocol was documented and registered in the International Prospective Register of Systematic Reviews (PROSPERO) (CRD42024527300). Ethical approval and participant consent were not required because we analysed data from published studies. Health Data Research United Kingdom’s (HDR-UK) Patient and Public Involvement and Engagement (PPI) were consulted on the conception, approach and search strategy.

### Search strategy and selection criteria

Embase and MEDLINE (via the OVID interface) were the bibliographic databases selected for the systematic review, as they provided the scientific literature in public health and biomedical sciences with the most relevant and ‘unique’ references.^20^^,^^21^

A preliminary search was conducted on Embase and MEDLINE on March 7, 2024, to validate the concept and prevent redundancy in research efforts.^22^ Once the search was completed, the results were reviewed to verify the concepts and ensure a sufficient collection of relevant studies for review. The search terms were refined for the next search and reviewed iteratively until the results accurately captured the research question and only included studies that covered search terms in the titles and abstracts, such as generic terms of the main exposures (economic, deprivation, employment) and observable asthma outcomes from diagnosed asthma patients (admissions, exacerbations and mortality). The search terms were finalised on April 3, 2025. The search terms included English language publications from January 1, 2010, to April 3, 2025. The search strategy was devised by ZG, HW and JKQ (clinician) (Supplementary material Table S2a–c).

The Population, Intervention, Comparator, Outcome, and Study (PICOS) framework was employed to formulate the eligibility criteria (Supplementary material Table S3).^23^ Outcome measures included binary, count, rates or time to (first) event, thus reporting odds ratio (OR), risk ratio (RR), incidence rate ratio (IRR) or hazard ratio (HR).

**Fig. 1:**
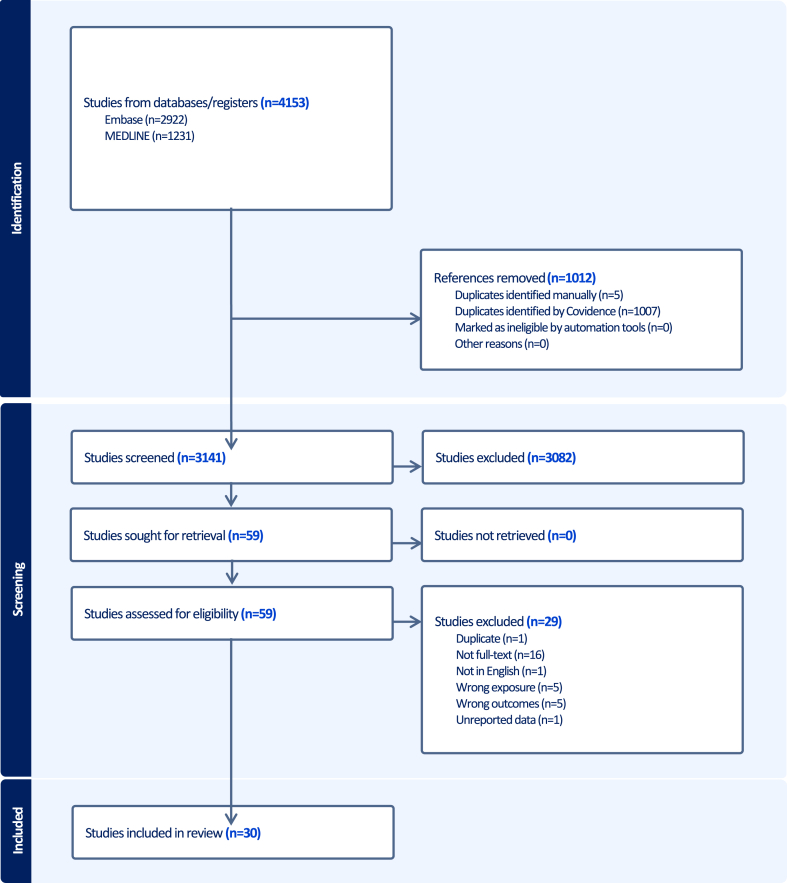
**PRISMA flow diagram of study screening and selection**.

The RIS files of the databases were exported to Covidence to automatically identify and remove duplicates for screening the titles and abstracts.^24^ To minimise the risk of error/bias, multiple independent reviewers (IK, EM, AMA, and AT) assessed the titles and abstracts of the retrieved studies according to the eligibility criteria. In cases of disagreement, a referee was consulted (HW). Then, the full text of the remaining studies was screened to determine its relevance to the research question and ensure that none of the exclusion criteria were present. Justification was provided for the excluded studies (Supplementary material Table S4). The PRISMA flow diagram illustrates all the steps in the selection process (Fig. 1).

Data from the full-text studies were extracted from Covidence to outline the study’s main characteristics, including exposure, population, outcomes, sociodemographic covariates, body mass index (BMI), smoking, and key findings (ZG, lK, EM, AMA, and AT). Any discrepancies were discussed between the two sets of initial reviewers, and where an agreement could not be reached, a referee was consulted (HW).

Three risk-of-bias tools for eligible studies were used depending on the study design. ROBINS-E was used to evaluate the risk of bias in observational studies.^25^^,^^26^ The Risk of Bias (RoB 2) was used for randomised control trials.^27^ For cross-sectional studies, an adapted RoB domains framework was used.^28^^,^^29^ After completing the domains, an overall judgement was made (low, moderate or high) and reported in the review. Two reviewers (ZG, HW) independently assessed the risk of bias in all included studies and completed the assessment tool in Word (Supplementary material Table S5a–c).

### Data analysis

Data were pooled using random-effects meta-analysis using Stata (version 18) based on comparable exposure, outcomes, covariates and methodology.^30^ Forest plots illustrated the heterogeneity between studies, where the I^2^ statistic >50% indicated a substantial impact of heterogeneity.^31^ Since odds ratios have favourable mathematical properties, meta-analyses were based on odds ratios.^32^

Ad-hoc transformations were applied to selected unit effect measures to allow comparisons based on a derivation of the conversion formula (Supplementary material Appendix S1).^33^^,^^34^ We estimated the baseline risk for studies reporting HR. In Cardet et al.,^35^ β coefficients were reported from the structural equation model predicting the direct association between low SES (latent variable) and asthma-related hospitalisations (0 or 1+). While the paper did not specify whether the structural equation model was probit or logit, it was more likely to be probit because the β coefficients are reported by standard deviation. From Amemiya’s study,^36^ probit estimates can be transformed to log odds by a factor of 1.6 and then exponentiated to obtain approximate odds ratios and 95% confidence intervals. Renzi-Lomholt et al.^37^ reported the odds ratio for exacerbations in the least deprived compared to the most deprived (reference category). In contrast, the other studies had the least deprived group as the reference category. For consistency, the odds ratio and 95% confidence interval were transformed by taking the inverse for quantitative aggregation.^33^^,^^38^

The overall effect size was estimated using a random-effects (RE) model. The weighting of the RE model was based on the inverse of the total variance. The estimator for between-study variability was based on the Restricted Maximum Likelihood (REML) method to obtain an unbiased, non-negative estimate of between-study variability.^39^ Subgroup analysis was also performed to explore heterogeneity between age-related population groups (patients below 18 compared to patients at least 18 years old).

### Role of the funding source

The funder of the study had no role in study design, data collection, data analysis, data interpretation, or writing of the report.

## Results

3632 studies were identified, 2583 from Embase and 1049 from MEDLINE (Fig. 1). After removing duplicates (1012 articles), 3141 articles were screened based on their titles and abstracts. 59 articles were included in the full-text screening, of which 29 were excluded (Supplementary material Table S4). Therefore, 30 studies were included in the systematic review and meta-analysis.^35^^,^^37^^,^^40^, ^41^, ^42^, ^43^, ^44^, ^45^, ^46^, ^47^, ^48^, ^49^, ^50^, ^51^, ^52^, ^53^, ^54^, ^55^, ^56^, ^57^, ^58^, ^59^, ^60^, ^61^, ^62^, ^63^, ^64^, ^65^, ^66^, ^67^

Studies were conducted in five different countries: USA (n = 18; 60%), UK (n = 7; 23%), Canada (n = 3, 10%), France (n = 1; 3%) and Denmark (n = 1; 3%) (Table 1). The publication dates of the included studies ranged from 2010 to 2025, with most papers published in 2024 (n = 9; 30%). Analyses were generally of observational design (n = 29; 97%), with cohort studies (retrospective, prospective or population-based) the most common. One study was both a cross-sectional and longitudinal study.^65^

**Table 1:** Study characteristics of the included studies.

Study	Country	Population	Study type	Main exposure(s)	Main outcome(s)	Variable(s)	Definition(s) or measurement(s)	Main findings^a^ (95% CI)
Disano et al. (2010)^43^	Canada	46,173 urban dissemination areas	Descriptive	Institut national de santé publique du Québec (INSPQ) Deprivation Index for health	Age-standardised hospital admissions rates for the treatment of ACSCs (COPD, diabetes and asthma in children)	Count	Number of ambulatory care sensitive conditions related hospital admissions	• Low INSPQ: 270 per 100,000 • High INSPQ: 161 per 100,000
Law et al. (2011)^55^	USA	238,678 adults	Cross-sectional (prevalence)	The ratio of family income to federal poverty level	Asthma-related emergency room or urgent care center visit in the past 12 months	Binary (yes/no)	Patient answered “yes” to the two questions: 1. “During the past 12 months, have you had an episode of asthma or an asthma attack?” 2. “During the past 12 months, have you had to visit an emergency room or urgent care center because of asthma?	OR = 1.32^a^ (1.03−1.68)
Ungar et al. (2011)^44^	Canada	490 children	Cohort (retrospective)	Child’s family socioeconomic and demographic characteristics	Asthma exacerbations (hospitalisations and emergency department visits)	Count	A count of urgent visits (hospitalizations and ED visits) during 1-year follow-up, where an ED visit that resulted in a hospital admission was counted as a single exacerbation	β = −0.33^a^ (−0.64/-0.014)
Auger et al. (2013)^56^	USA	601 children	Cohort (prospective)	Total annual household income	(Time to) readmission for an acute asthma exacerbation	Event time	Admission diagnosis recorded as acute asthma exacerbation with evidence based clinical pathway for acute asthma care by the admitting physician.	HR = 1.82^a^ (0.78–4.23)
To et al. (2014)^41^	Canada	Asthma prevalent population (unspecified)	Cohort (population-based)	Ontario Marginalisation Index (ON-Marg) (deprivation quintiles)	Asthma-specific (or underlying) mortality	Count	• Asthma-specific mortality: cases with asthma listed as the primary cause of death determined via International Classification of Diseases (ICD-9) codes • Asthma-contributing mortality: one of the secondary causes of death determined via International Classification of Diseases (ICD-9) codes	Asthma-underlying or specific mortality: • Poisson Rate Ratio = 1.60^a^ (1.16–2.20) Asthma-contributing mortality: • Poisson Rate Ratio = 1.34^a^ (1.10–1.64)
Zhang et al. (2017)^42^	USA	5535 children (aged 2–17)	Cohort	Income (Low: <$35,000, Middle: $35,000–$75,000, High: ≥$75,000)	Emergency room (ER) visits (self-reported measures of one or more ED visits in the past 12 month)	Binary (yes/no)	ED visit status was recorded in response to: “During the past 12 months, has {child’s name} had to visit an emergency room or urgent care center because of {his/her} asthma?”	Prevalence ratios = 0.47^a^ (0.28–0.80)
Gupta et al. (2018)^57^	England (UK)	14,830 recorded asthma deaths of children and adults 542,877 emergency asthma admissions over the age of 5	Cross-sectional	English IMD	Asthma mortality and hospital admissions	Count (hospital admissions and mortality)	• The numbers of registered deaths in England with underlying cause of asthma (International Classification of Diseases Version 10 (ICD-10) J45 and J46) recorded on the death certificate. • Numbers of emergency admissions with a primary diagnosis of asthma	Mortality: • 5–44: IRR = 0.81^a^ (0.69–0.96) • 45–74: IRR = 1.37^a^ (1.24–1.52) • 75+: IRR = 1.30^a^ (1.22–1.39) Admissions: • 5–44: IRR = 3.34^a^ (3.30–3.38) • 45–74: IRR = 2.01^a^ (1.98–2.05) • 75+: IRR = 1.43^a^ (1.39–1.47)
Mazalovic et al. (2018)^45^	France	255 children and adult patients	Cohort (ancillary, prospective)	SES (derived from the French National claims database and GPs computerised questionnaires)	Asthma exacerbations (followed by measures on managing asthma exacerbations)	Count	The occurrence of at least one of the following asthma-related events: OCs courses, unplanned medical visits to a GP or a hospital emergency department, hospitalization, or death	• Mann–Whitney test: p = 0.38 • OR = 0.27 (0.09–0.84)
Cardet et al. (2018)^40^	USA	381 adult participants	RCT	SES correlates: (1) Income group (low: <$50,000 vs. high) (2) Household educational level (<Bachelor’s Degree) (3) Perceived Stress Level (≥20)	Asthma exacerbations (requiring systemic corticosteroids)	Count	Satisfying both the treatment failure criteria and ≥1 of the following: • Failure to respond to rescue algorithm within 48 h • FEV1 ≤50% of baseline or <40% of predicted (2 consecutive measurements) • Levalbuterol use of ≥16 puffs/day for 48 h • Exacerbation per physician opinion • Systemic corticosteroid treatment for asthma *NB: The treatment failure is defined as ≥1 of the following:* • *Peak expiratory flow ≤65% of baseline (2 of 3 consecutive measurements)* • *FEV1 ≤80% of baseline (2 consecutive measurements)* • *Levalbuterol dose increase by ≥8 puffs/day for 48 h (vs. baseline)* • *Additional ICS or systemic corticosteroid treatment* • *Asthma-related emergency department visit or hospitalization with systemic corticosteroid treatment* • *Participant dissatisfaction with treatment* • *Physician clinical safety judgment*	Poisson Rate Ratio = 1.80^a^ (1.10–3.10)
Grunwell et al. (2018)^46^	USA	579 children (6–18 years old)	Cohort (retrospective)	Income (below and above the poverty line)	PICU (Paediatric Intensive Care Unit) admission	Binary (yes/no)	Admitted to the hospital for an asthma exacerbation in the last 12 months	OR = 1.28^a^ (1.02–1.61)
Eum et al. (2019)^47^	USA	2093 children (2011: 1070 & 2015: 1023)	Cross-sectional	SES (median household income, residents’ education level, health insurance coverage, and unemployment rate)	Children’s asthma-related emergency department (ED) utilisation	Count	The total count of daily pediatric asthma-related ED visits based on primary diagnosis code (International Classification of Diseases, 9th Revision (ICD-9) 493; ICD-10 J45)	Median household income: • 2011: RR = −0.03^a^ (−0.04/−0.02) • 2015: RR = −0.03^a^ (−0.03/−0.02) Unemployment: • 2011:RR = −0.01^a^(−0.04–0.03) • 2015: RR = −0.01^a^ (−0.04–0.03)
Seibert et al. (2019)^48^	USA	342 adults (18–41 years old)	Cohort (longitudinal)	Self-reported household income category (<$15,000, $15,000–$29,999, $30,000–$50,000, >$50,000)	Asthma-related emergency department (ED) visits and hospitalization	Binary (yes/no)	Same-day care (ED visit, hospitalization, or any same-day medical visit such as a walk-in clinic or urgent care center) in the previous 3 months	Asthma-related ED visit: • OR = 0.88^a^ (0.80–0.97) Asthma hospitalization: • OR = 0.94^a^ (0.82–1.08)
Brite et al. (2020)^67^	USA	30,452 adults	Cohort	SES defined as (1) Education and income and (2) race/ethnicity	Asthma-related emergency department (ED) visits	Count	The number of asthma-related ED visits determined by the International Classification of Diseases, Ninth Revision, Clinical Modification (ICD-9-CM) and International Statistical Classification of Diseases, Tenth Revision, Clinical Modification (ICD-10-CM) codes as a principal diagnosis or a respiratory condition listed as the principal diagnosis and asthma listed as a secondary diagnosis	β = 11.70 (10.60–12.70)
Molina et al. (2020)^54^	USA	664 children	Cross-sectional (retrospective)	Alabama area deprivation index (proxy for neighbourhood deprivation)	Severe hospitalization (requiring continuous albuterol or intensive care unit care)	Binary (yes/no)	Intensive care unit care or continuous albuterol	OR = 1.09^a^ (0.73–1.63)
Jroundi & Tse (2021)^49^	USA	66,835 children	Cohort (retrospective)	State median household income (quartiles)	Time to asthma-related readmission and time to first asthma-related ED visit date	Event time	The first asthma-related readmission after the hospitalization discharge date	HR = 1.33^a^ (1.15–1.53)
Alsallakh et al. (2021)^50^	Wales (UK)	Main study cohort: 106,926 children and adults Asthma mortality analysis: 327,906	Cohort	Welsh IMD quintiles	Asthma-related health service utilisation and asthma-related deaths	Count (hospital admissions and mortality)	Patients with a primary diagnosis of asthma (J45) or status asthmaticus (J46) coded using the 10th Revision of the International Classification of Diseases (ICD-10). Among these, emergency admissions were defined as coming via A&E departments, urgent referrals from GPs, consultant clinics, bed bureaus, or NHS Direct	Asthma-related accident and emergency attendances: • IRR = 1.27^a^ (1.10–1.46) Asthma emergency admissions: • IRR = 1.56^a^ (1.39–1.76) Asthma-related death: • RR = 1.56^a^ (1.18–2.07).
Busby et al. (2021)^51^	UK	127,040 adult patients	Cohort (population-based)	Indices of Multiple Deprivation of their general practice (a proxy measure for individual SES)	Asthma presentation, processes and healthcare outcomes, including exacerbations	Binary (yes/no)	Read code indicating an ‘Asthma Exacerbation’ or ‘Asthma Attack, prescription of acute oral corticosteroids (OCS), or a lower respiratory infection requiring antibiotics	OR = 1.27^a^ (1.13–1.42)
Cardet et al. (2022)^35^	USA (including Puerto Rico)	990 adults	Cross-sectional (ancillary)	SES (based on a multidomain, latent variable defined by poverty, education, and unemployment)	Asthma morbidity (exacerbations and Asthma Control Test score)	Binary (yes/no)	Outpatient corticosteroid bursts for asthma, emergency room [ER]/urgent care [UC] visits and hospitalizations	Low SES and hospitalizations: • β = 0.24^a^ (0.11–0.38) Low SES and asthma ER/UC visits: • β = 0.03^a^ (0.00–0.05) Poverty and additional asthma hospitalisations: • β = 0.48^a^ (0.15–0.80) Unemployment and ER/UC visit: • β = 0.03^a^ (0.01–0.06)
Mukherjee et al. (2022)^52^	England (UK)	2110 children	Observational	English IMD	Healthcare resource utilisation and severity, including admissions and deaths	Count (hospital admissions and mortality)	Number of deaths in PICU	β = 1.28^a^ (1.10–1.49)
Kallis et al. (2023)^59^	England (UK)	898,763 adults (training sample)	Cohort	English IMD	Asthma exacerbation (n = 93,625)	Binary (yes/no)	At least one asthma exacerbation recorded within 90 days from their study start	OR = 1.06^a^ (1.04–1.09)
Renzi-Lomholt et al. (2024)^37^	Denmark	29,851 children	Cohort	4 markers of parental socio-economic position (workforce attachment, family disposable income, family highest level of attained education and metropolitan residence)	Uncontrolled, exacerbating, and severe asthma	Binary (yes/no)	Redemption of at least 187.5 mg of prednisolone, severe as asthma related (ICD-10 codes J45, J46, J96, J960 or J969) hospitalisation and near-fatal as intensive care admission with the ICD-10 codes	OR = 0.68^a^ (0.58–0.79)
Khalaf et al. (2024)^53^	England, UK	119,611 children	Cohort	English IMD	Asthma exacerbation (general practitioner (GP)-managed (short course of oral corticosteroids (OCS)) or hospital-managed (A&E visit or hospital admission for asthma))	Event time	General practitioner (GP)-managed (short course of oral corticosteroids (OCS)) or hospital-managed (A&E visit or hospital admission for asthma, using International Classification of Disease (ICD)-10 codes J45 and J46	• Age 5–11.9 years: HR = 1.20^a^ (1.20–1.30) • Age 12–15.9 years: HR = 1.40^a^ (1.20–1.50) • Adolescents: HR = 1.30^a^ (1.20–1.50)
Simms-Williams et al. (2024)^58^	UK	1,385,326 children and adults	Cohort	IMD	Asthma-related hospital and intensive care unit (ICU) admissions	Count	Hospital admissions with associated ICD-10 asthma diagnosis codes J45 and J46 as the primary diagnostic code for the admission	Asthma-related hospital admissions: • 5–11 years: IRR = 1.51^a^ (1.30–1.75) • 2–17 years: IRR = 1.52^a^ (1.22–3.34) • 8+ years: IRR = 1.43^a^ (1.33–1.54) Asthma-related ICU admissions: • 5–11 years: IRR = 1.98^a^ (1.03–3.79) • 12–17 years: IRR = 2.09^a^ (0.88–4.98)^a^ • 18+ years: IRR = 1.35^a^ (0.96–1.89)
Akinyemi et al. (2024)^60^	USA	1,665,516 adults	Retrospective cross-sectional study	Distressed Communities Index (DCI)	Asthma-related ED visits	Binary (yes/no)	The occurrence of asthma-related ED visits based on diagnostic codes and records indicating asthma-related complaints and treatment	OR = 1.65^a^ (1.62–1.69)
Gaietto et al. (2024)^65^	Puerto Rico (USA)	209 ‘youths’ (6–14 and 9–20)	Cross-sectional and longitudinal study	Low annual household income (<$15,000 per year)	Recurrent severe asthma exacerbation based on asthma-related ED visits and hospitalisation	Binary (yes/no)	Visit to the emergency department (ED) or urgent care for asthma or a hospitalization for asthma	OR = 12.25^a^ (2.59–57.97)
Scott et al. (2024)^61^	USA	211 individuals	Community-based, cross-sectional study	Perceived financial status, income and occupation	Exacerbation-based (un)controlled asthma: Uncontrolled asthma: ≥2 asthma exacerbations in the past year Controlled asthma: ≤1 or no exacerbations	Binary (yes/no)	Patient answered “yes” to question 1: 1. During the past 12 months, have you had an episode of asthma or an asthma attack?” And reported ≥2/year to question 2: 2. “During the past 12 months, how many asthma episodes or attacks have you had?”	Occupation: • OR = 0.69^a^ (0.23–2.05) Perceived financial status: • OR = 0.81^a^ (0.27–2.50)
Khalid et al. (2024)^62^	USA	423,140 adult asthma admissions	Retrospective observational study	National quartile for household income	In-hospital mortality (per admission)	Count	Number of deaths in hospital per asthma admission	OR = 0.77^a^ (0.51–1.15)
Skeen et al. (2024)^66^	USA	193 children	Cross-sectional study	Child Opportunity Index (COI) 2.0	Exacerbation-prone asthma	Binary (yes/no)	An asthma-related event requiring an unscheduled visit to an emergency department or urgent care facility, overnight hospitalization, and/or course of oral or injectable corticosteroids	COI overall: • OR = 1.19^a^ (0.92–1.54) Household income ($5000 increments): • OR = 1.00^a^ (0.89–1.12)
Xu et al. (2024)^64^	USA	198,873 adults	Retrospective cohort study	Neighborhood Deprivation Index (NDI)	Acute asthma exacerbation (AAE) and asthma-related emergency department and urgent care (ED/UC) visits in adults with mild asthma.	Count	1. A hospitalization, ED visit, or hospital-based observation with: • A principal discharge diagnosis of asthma or wheezing OR • Specific respiratory conditions being the principal or primary diagnosis, and with either exacerbation or status asthmaticus being the secondary diagnosis 2. A systemic corticosteroid administration in which asthma was the principal or primary encounter diagnosis code or was associated with the systemic corticosteroid order	Acute asthma exacerbation: • RR = 1.27^a^ (1.20–1.35) Asthma-related ED visits: • RR = 1.75^a^ (1.62–1.88)
Miller et al. (2025)^63^	USA (including Puerto Rico)	15,877 children	Cohort study	Child Opportunity Index (COI)	Asthma with Recurrent Exacerbations	Count	At least two reports of systemic corticosteroid use at any time during the entire follow-up period with each considered an exacerbation event if separated by a minimum of 30 days	Overall COI: • IRR = 1.26^a^ (0.99–1.59) Social and economic domain categories of COI: • IRR = 1.22^a^ (0.97–1.53)

OR = odds ratio. HR = hazard ratio. IRR = incidence rate ratio. RR = risk ratio. β = regression coefficient.

a Adjusted.

Studies either had measured income per se as an exposure (n = 15; 50%) or as part of a deprivation index (n = 13; 43%), such as IMD, WIMD, Neighborhood Deprivation Index (NDI), Distressed Communities Index (DCI), Institut national de santé publique du Québec (INSPQ) Deprivation Index and Ontario Marginalization Index (ON-Marg). The remaining two studies measured poverty^35^ or perceived financial status.^61^ One study assessed the relationship between SES and asthma-related emergency department (ED) visits.^67^ In addition to income, three studies (10%) measured employment per se as an exposure and were included in the narrative synthesis.^35^^,^^47^^,^^61^

The sample size varied between 193 and 1,665,516 patients.^60^^,^^66^ The percentage of females in the studies ranged from 37% to 83.9%.^35^^,^^54^ The mean and median age were commonly reported, with similar highest ages: 52.1 years (mean)^51^ and 53 years (median).^59^ Of the studies that measured ethnicity, white was the largest ethnic group, reaching as high as 97.5%.^51^ Studies that reported the BMI categories had most participants in the normal range.^42^^,^^45^^,^^46^^,^^53^^,^^58^ However, studies reporting the average BMI were between overweight and obese BMI, ranging from 27.8 to 32.3.^40^^,^^51^ Most patients were non- or never-smokers.^35^^,^^45^^,^^48^^,^^51^^,^^58^^,^^64^

19 studies (63%) investigated the association between income as a proxy for SES, deprivation or poverty, and exacerbations.^35^^,^^37^^,^^40^^,^^44^, ^45^, ^46^^,^^48^^,^^49^^,^^51^^,^^53^, ^54^, ^55^, ^56^^,^^59^^,^^61^^,^^63^, ^64^, ^65^, ^66^ Generally, studies defined exacerbations by ED visits/hospitalisations or oral corticosteroids (OCS)/hospitalisations, though some studies used both.^53^^,^^64^^,^^66^ Multiple studies defined exacerbations by prescription only: acute OCS,^35^ systemic corticosteroid,^63^ and OCS or lower respiratory infection requiring antibiotics.^51^ Three studies included the following definitions: failure to meet treatment criteria of levalbuterol or systemic corticosteroid and diagnostic tests,^40^ medical contact and death,^45^ and intensive care admissions and prednisolone.^37^ Eight (%) included physician-defined exacerbations,^37^^,^^46^^,^^49^^,^^51^^,^^54^^,^^56^^,^^59^^,^^63^^,^^66^ and six studies (%) relied on self-reported exacerbations.^35^^,^^40^^,^^45^^,^^48^^,^^55^^,^^61^ One study specified physician-defined and hospital-managed exacerbations.^53^ One study identified risk factors associated with recurrent severe asthma exacerbations from two visits, by exploring the possible permutations to see whether both the risk factor and outcome changed or ‘persisted’ in both visits.^65^ Depending on the study methods, exacerbations were mainly measured as a binary outcome (yes/no) and reported ORs. Other studies reported exponentiated Poisson coefficients and rate ratios for the exacerbation risk,^44^^,^^64^ HRs for time to exacerbation,^53^^,^^56^ and IRRs for childhood asthma with recurrent exacerbations.^63^

Five studies (23%) investigated the impact of SES on the number of hospital admissions.^43^^,^^50^^,^^52^^,^^57^^,^^58^ General and specific definitions were applied. Alsallakh et al.^50^ included asthma admissions per se to distinguish them from emergency admissions, which were defined as “coming via A&E departments, urgent referrals from GPs, consultant clinics, bed bureaus, or NHS Direct”. Similarly, Simms-Williams et al.^58^ had asthma admissions as a primary outcome and asthma-related ICU admissions as a secondary outcome. Disano et al.^43^ and Gupta et al.^57^ provided a general definition of hospital admissions, while Mukherjee et al.^52^ focussed on the number of paediatric intensive care unit (PICU) admissions. Three of the five studies reported age-standardised rates.^43^^,^^52^^,^^57^

Five studies (23%) examined the association between SES and asthma mortality.^41^^,^^50^^,^^52^^,^^57^^,^^62^ Three studies used ICD-9^41^ and ICD-10 codes^50^^,^^57^ to identify asthma as the underlying cause of death. To et al.^41^ further established asthma as a secondary cause, defined as asthma-contributing mortality, and Alsallakh et al.^50^ also included deaths with any mention of asthma in the definition of mortality. Mukherjee et al.^52^ based mortality in PICU on a prediction score from a model known as the Paediatric Index of Mortality version 2 (PIM2). While assessing seasonal variations and differential outcomes in adult admissions for asthma, Khalid et al.^62^ reported the association between income and in-hospital mortality using the US National Inpatient Sample database.

Four studies (13%) had more than one exposure and/or outcome, thus reporting multiple associations.^50^^,^^52^^,^^57^^,^^64^ Further information on the study covariates and outcomes, including effect sizes and measures, is provided in Supplementary material Table S6a–d.

Most of the studies were assessed using the ROBINS-E tool (n = 19; 63%). Four studies (13%) were deemed at high risk of bias due to confounding (not adjusting for confounding or persistent residual confounding) and selection bias (Table 2). Most studies had a low risk of bias, with uncontrolled or residual confounding as the most common bias. Most studies adjusted for at least two of the three prespecified confounders (age, sex/gender or ethnicity), with seven (%) adjusting for all.^35^^,^^49^^,^^54^^,^^58^, ^59^, ^60^^,^^62^^,^^66^ Gaietto et al.^65^ was assessed using the ROBINS-E tool given the outcome of interest, i.e., the association between a ‘persistent’ risk factor (income) and ‘persistent’ recurrent severe asthma exacerbations with persistent defined as having a risk factor and outcome in both visits 1 and visits 2, was reported in the longitudinal model.

**Table 2:** Summary of risk of bias in eligible studies.

Study	Risk of Bias tool	Risk	Bias
Disano et al. (2010)^43^	RoB (adapted)	High	Did not adjust for confounding
Law et al. (2011)^55^	RoB (adapted)	Low	Missingness and recall bias from survey data
Ungar et al. (2011)^44^	ROBINS-E	Moderate	Uncontrolled or residual confounding and reporting bias
Auger et al. (2013)^56^	ROBINS-E	Low	Uncontrolled or residual confounding
To et al. (2014)^41^	ROBINS-E	Low	Uncontrolled or residual confounding
Zhang et al. (2017)^42^	ROBINS-E	High	Uncontrolled or residual confounding
Cardet et al. (2018)^40^	RoB 2	Low	N/A
Grunwell et al. (2018)^46^	ROBINS-E	Low	Uncontrolled or residual confounding
Mazalovic et al. (2018)^45^	ROBINS-E	High	Did not adjust for confounding
Gupta et al. (2018)^57^	RoB (adapted)	Low	Uncontrolled or residual confounding
Seibert et al. (2019)^48^	ROBINS-E	Low	Uncontrolled or residual confounding
Molina et al. (2019)^54^	RoB (adapted)	Low	Selection bias
Eum et al. (2019)^47^	RoB (adapted)	Low	Uncontrolled or residual confounding
Brite et al. (2020)^67^	ROBINS-E	Low	Uncontrolled or residual confounding
Alsallakh et al. (2021)^50^	ROBINS-E	Low	Uncontrolled or residual confounding
Jroundi & Tse (2021)^49^	ROBINS-E	Low	Uncontrolled or residual confounding
Busby et al. (2021)^51^	ROBINS-E	Low	Uncontrolled or residual confounding and regression dilution
Mukherjee et al. (2022)^52^	RoB (adapted)	Low	Uncontrolled or residual confounding
Cardet et al. (2022)^35^	RoB (adapted)	Moderate	Selection bias and missingness
Kallis et al. (2023)^59^	ROBINS-E	Low	Uncontrolled or residual confounding
Renzi-Lomholt et al. (2024)^37^	ROBINS-E	Low	Uncontrolled or residual confounding
Khalaf et al. (2024)^53^	ROBINS-E	Moderate	Uncontrolled or residual confounding and missingness
Simms-Willliams et al. (2024)^58^	ROBINS-E	Low	Uncontrolled or residual confounding
Akinyemi et al. (2024)^60^	RoB (adapted)	Moderate	Uncontrolled or residual confounding and reporting bias
Gaietto et al. (2024)^65^	ROBINS-E	Low	Selection bias
Scott et al. (2024)^61^	RoB (adapted)	High	Selection bias and other bias (internal validity concerns)
Khalid et al. (2024)^62^	ROBINS-E	Low	Uncontrolled or residual confounding
Skeen et al. (2024)^66^	RoB (adapted)	Low	Uncontrolled or residual confounding
Xu et al. (2024)^64^	ROBINS-E	Low	Uncontrolled or residual confounding
Miller et al. (2025)^63^	ROBINS-E	Moderate	Uncontrolled or residual confounding and selection or temporal bias

Ten of the 19 studies were eligible for a meta-analysis, with half contributing to each population group (Table 3). Seven studies reported ORs, two reported HRs, and one reported β coefficients. The remaining studies had results that were not comparable in terms of age,^53^^,^^65^^,^^66^ exposure,^61^ effect measure,^40^^,^^44^^,^^63^^,^^64^ and lack of covariate adjustment.^45^

**Table 3:** Studies included for meta-analysis with the absolute, original and final/transformed estimates.

Study	n/N	Original estimate (95% CI)	Population group	Final/transformed estimate (95% CI)
Law et al. (2011)^55^	2367/238,678	OR = 1.32 (1.03–1.68)	Adults	OR = 1.32 (1.03–1.68)
Auger et al. (2013)^56^	135/601	HR = 1.82 (0.78–4.23)	Children	OR = 1.92 (0.77–5.24)
Grunwell et al. (2018)^46^	170/579	OR = 1.28 (1.02–1.61)	Children	OR = 1.28 (1.02–1.61)
Seibert et al. (2019)^48^	N/A	OR = 0.94 (0.82–1.08)	Adults	OR = 1.06 (0.93–1.22)
Molina et al. (2020)^54^	149/664	OR = 1.09 (0.73–1.63)	Children	OR = 1.09 (0.73–1.63)
Busby et al. (2021)^51^	5732/127,040	OR = 1.27 (1.13–1.42)	Adults	OR = 1.27 (1.13–1.42)
Jroundi & Tse (2021)^49^	9377/66,835	HR = 1.33 (1.15–1.53)	Children	OR = 1.37 (1.17–1.61)
Cardet et al. (2022)^35^	596/990	β = 0.24 (0.11–0.38)	Adults	OR = 1.47 (1.19–1.84)
Kallis et al. (2023)^59^	93,625/805,138	OR = 1.06 (1.04–1.09)	Adults	OR = 1.06 (1.04–1.09)
Renzi-Lomholt et al. (2024)^37^	2353/29,851	OR = 0.68 (0.58–0.79)	Children	OR = 1.47 (1.27–1.72)

The odds ratios presented in the final or transformed estimates originate from binary (yes/no) variables (8 studies) or event time variables (2 studies). Binary (yes/no) variables reported odds ratios (7 studies) and regression coefficients (1 study). Event time variables reported hazard ratios (2 studies).

OR = odds ratio. HR = hazard ratio. β = regression coefficient. n = number of exacerbations. N = number of patients.

**Fig. 2:**
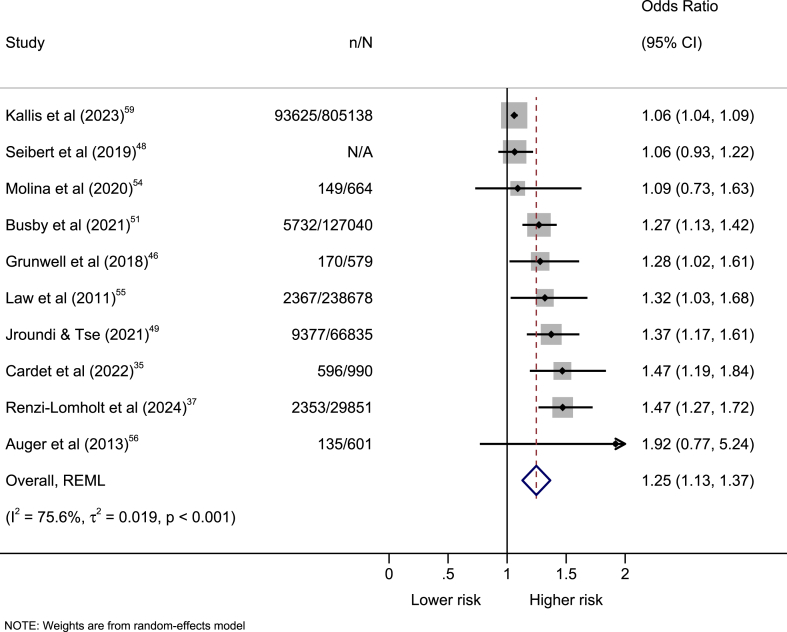
**Forest plot of the association between income and exacerbations.** Odds ratios are presented with 95% confidence intervals. The odds ratios presented in the original studies originate from binary (yes/no) variables (8 studies) or event time variables (2 studies). The red vertical line is the average effect estimate. The black vertical line shows no effect. OR = odds ratio. n = number of exacerbations. N = number of patients.

The lowest income group were more likely to be hospitalised or (re)admitted due to an exacerbation than the highest income group, albeit with substantial heterogeneity between studies (OR 1.25 [95% CI 1.13–1.37]; I^2^ = 75.6%) (Fig. 2). We reject the homogeneity test of study-specific effect sizes (p < 0.001). Therefore, we can infer significant heterogeneity between the individual studies. The significance test (p < 0.001) suggested that the overall effect size is statistically significantly different from zero.

**Fig. 3:**
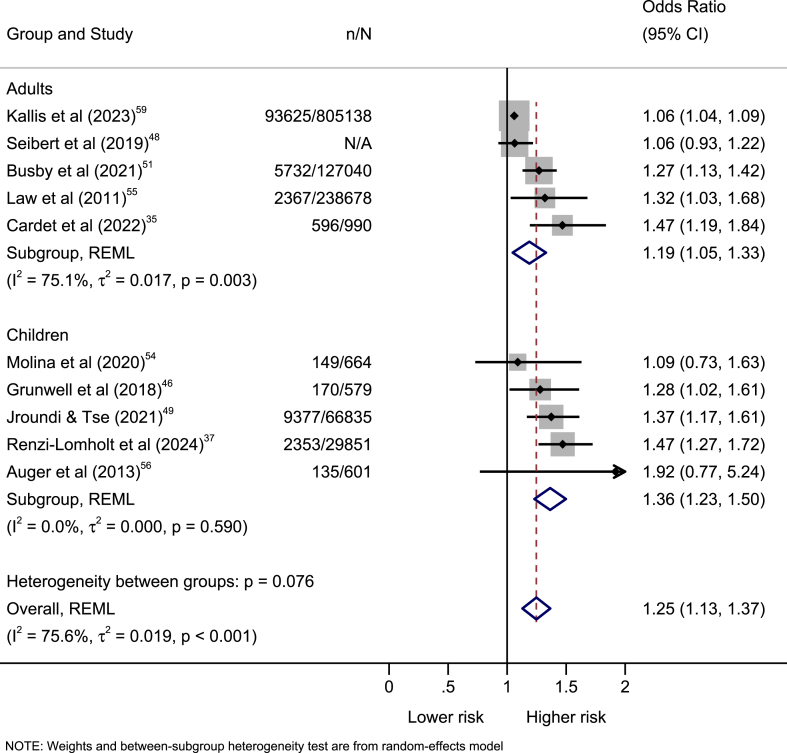
**Forest plot of the association between income and exacerbations by population group (children and adults).** Odds ratios are presented with 95% confidence intervals. The odds ratios presented in the original studies originate from binary (yes/no) variables (8 studies) or event time variables (2 studies). The red vertical line is the average effect estimate. The black vertical line shows no effect. OR = odds ratio. n = number of exacerbations. N = number of patients.

The subgroup analysis showed no difference in the association between children (1.36 [1.23–1.50]; I2 = 0.0%) and adults (1.19 [1.05–1.33]; I2 = 75.1%) (Fig. 3). With the potential impact of heterogeneity greater in adults than in children, we can infer that studies with adults correspond to most of the variability of the effect sizes. The test of group differences indicated no statistically significant subgroup effect (p = 0.076), meaning that the population group does not modify the effect of income on exacerbation.

Two studies investigated the association between employment and EC attendance or ED utilisation, using unemployment as an indicator or component of SES.^35^^,^^47^ The first study examined the pathways and identified mediating factors in African American/Black and Hispanic/Latinx adults with moderate to severe asthma.^35^ Unemployment showed stress-mediated indirect associations with increased healthcare utilisation. Those living in poverty, who were more likely to be unemployed (73.7% vs. 40.8%; p < 0.001), experienced higher rates of asthma hospitalisations (23.6% vs. 14.0%; p < 0.001). Notably, unemployment was the only SES measure showing stress-mediated indirect associations with emergency room/urgent care visits (β 0.03 [95% CI 0.01–0.06]).

The second study was a cross-sectional study on a paediatric asthma cohort in New York.^47^ Children living in areas where regional economic development projects were completed during the study period (2011–2015) had lower ED utilisation. The unemployment rate was used as an indicator for the latent SES variable in a spatial regression analysis to account for geographic variation and local economic conditions. While there was a significant decrease in ED utilisation in areas where economic development projects were completed (β_difference-in-differences_-1.526 [SE = 0.686]), this was not directly linked to changes in unemployment rates. The relative risk for unemployment was not statistically significant in 2011 (RR -0.01 [95% CI −0.04–0.03]) and 2015 (−0.01 [−0.04–0.03]), suggesting no evidence of an association between unemployment and asthma-related ED utilisation. However, other SES indicators, namely median household income and health insurance coverage, were key socioeconomic predictors of children’s asthma-related ED utilisation. While unemployment was significantly associated with asthma-related EC utilisation in adult populations, mainly through stress-mediated pathways, its association with paediatric asthma outcomes is less clear compared with other indicators.

One study examined whether employment was associated with uncontrolled asthma based on having two or more exacerbations in the previous year.^61^ Based in rural Appalachia, Kentucky, this cross-sectional study measured employment using employment status. While employment appeared to impact whether an individual reported having asthma, it was not a statistically significant predictor of uncontrolled asthma after controlling for age, sex, perceived financial status, and accommodation (OR 0.69 [0.23–2.05]). Given the broad categorisation, where unemployment was in the same group as part-time employment, full-time students and retired, it is hard to determine the role of unemployment in predicting exacerbation-based uncontrolled asthma.

## Discussion

Overall, we found a clear association between lower income and exacerbations in both children and adults. We were unable to assess statistically the association between unemployment and asthma outcomes; instead, we narratively synthesised the role of unemployment. Despite the limited evidence, narratively, we found that unemployment was associated with ED visits. However, the association between unemployment and uncontrolled asthma based on exacerbations was unclear.

The narrative synthesis indicated a stress-mediated indirect association between unemployment and asthma-related healthcare utilisation. This suggests that the psychological burden of unemployment may exacerbate asthma symptoms and hinder effective self-management. Evidence indicates that sustained physiological stress intensifies the immune response, such as hypothalamic-pituitary-adrenocortical activation.^68^, ^69^, ^70^, ^71^ This leads to a decrease in β_2_ adrenergic and glucocorticoid receptors, which reduces the responsiveness to asthma medication and, in turn, increases the risk of exacerbation.^70^^,^^72^ Employment provides financial stability and benefits, such as employer health insurance and sick leave, that can facilitate disease management.^73^ Without these privileges, the unemployed are likely to delay or forgo health care-seeking behaviours and present themselves in ED with more severe outcomes.^7^^,^^74^ However, this is limited to adults as unemployment is not a strong predictor of paediatric asthma outcomes compared with other SES indicators.

The wider literature on income and asthma corroborates our findings. Low-income households are strongly associated with limited access to essential resources and amenities, including nutritious food and healthcare services, that increase their risk of asthma exacerbations.^75^, ^76^, ^77^ They are more likely to live in subsidised or social housing, which is often too small and of poor quality, beset with overcrowding and disproportionate levels of indoor and outdoor pollution.^78^ Maintaining and repairing these homes is expensive, thus increasing their exposure to damp, mould, pests, and second-hand smoke. Unsurprisingly, subsidised housing is associated with experiencing asthma attacks in the previous year.^79^ Children are at greater risk due to their limited immune response to microbial exposure and more so in urban areas whose greater residential density increases the concentration of these agents.^80^^,^^81^ Also, children with asthma living in these settings are at greater odds of an ED visit than children residing with homeowning parents.^82^ As a result, asthma patients on low incomes are denied the structural means to ameliorate their condition. They may also struggle to afford medication.

Pooled estimates from six high-income countries indicated that lower household income in early childhood was associated with the risk of developing poorer asthma-related outcomes.^83^ A study investigating the association between socioeconomic position (SEP), an indicator of SES,^84^^,^^85^ and asthma in a historical cohort of male university students found an association between low SEP in early life and asthma.^86^ However, there is no association between adult SEP and adult-onset asthma. Using a different measure of SES, such as IMD, would possibly yield different results. Notwithstanding the smaller magnitude, our findings align with previous systematic reviews and meta-analyses on socioeconomic status and asthma outcomes. Lower SEP was associated with higher rates of asthma-related ED attendances.^15^ A systematic review identified 31 studies where the lowest-income group had between 1.5 and 5 times the hospitalisation rate for asthma compared with the highest-income group.^87^ Unlike these reviews, this review conducted a subgroup analysis of children and adults to explore heterogeneity by population. While there was a clear association in both children and adults, there was no evidence of a significant difference in the magnitudes of effect.

Other factors, such as medication use and adherence (or lack thereof), may relate directly to poor asthma outcomes. There is a tendency to overuse short-acting β-2 agonists (SABA), which do not address inflammation but only offer immediate symptom relief. High SABA use is found to be associated with poorer clinical outcomes, including exacerbations,^88^^,^^89^ hospitalisations,^90^^,^^91^ and mortality.^92^^,^^93^ Evidence illustrates a dose–response relationship between SABA inhaler use and exacerbation risk, with even mild asthma patients experiencing exacerbations, whose risk was further elevated with high SABA use.^94^, ^95^, ^96^, ^97^, ^98^ Many studies have demonstrated the benefits of high adherence to asthma controller therapy in reducing the risk of exacerbation and death.^99^, ^100^, ^101^, ^102^ However, the effects of inhaled corticosteroids (ICS) adherence on mainly exacerbations are less clear. Studies identified a non-linear, U-shaped association between ICS adherence and exacerbations.^103^, ^104^, ^105^, ^106^ In other words, better adherence does not always mean better asthma outcomes. Analysis from England observed that ICS adherence was better in Clinical Commissioning Group regions with greater socioeconomic deprivation, with these areas also having worse asthma outcomes which may relate to SES factors specifically.^107^ Also, the relationship between socioeconomic deprivation and asthma outcomes did not appear to be primarily mediated by poor adherence to ICS. SABA over-reliance and ICS adherence could be driven by suboptimal patient knowledge about the difference between maintenance and reliever medication, the patient’s need for immediate symptom relief, concerns about the side effects of steroids, and poor communication between patients and physicians as well as different prescribing behaviours by physicians due to perverse incentives.^108^

The main strength of this review was summarising the evidence on the effect of income and, though limited, employment, the most weighted IMD domains, on asthma outcomes. Both the meta-analysis and narrative synthesis offer a better understanding of the association between these domains and asthma outcomes. The studies included in this review were published between 2010 and 2025, thus reflecting more recent evidence than in previous reviews.^14^^,^^15^

However, there are limitations to this review. 60% of the included studies were from the US (18 studies) compared to seven from the UK, three from Canada and only two from the other European countries (Denmark and France). There are likely to be different constructs to assess SES. On the one hand, some North American studies used state^49^^,^^65^ or federal^40^^,^^55^^,^^62^ income thresholds. Other studies included stress,^40^ health insurance coverage,^47^ and race/ethnicity.^67^ Depending on the population, studies also looked at bespoke deprivation indices, such as the Child Opportunity Index (COI),^63^^,^^66^ Distressed Communities Index,^60^ and Neighborhood Deprivation Index.^64^ On the other hand, UK-based studies predominantly used IMD.^50^, ^51^, ^52^, ^53^^,^^57^, ^58^, ^59^ Not only does this affect the generalisability of findings to other Western countries but also many non-Western countries where there are greater inequalities. Only studies published in English were included for practical reasons, potentially introducing a language bias. We were unable to search in other language databases and, therefore, may have missed potentially useful studies.

Moreover, there are some limitations to the evidence. Due to the lack of studies available to conduct a meta-analysis, we narratively synthesised the role of unemployment on asthma outcomes. However, this was limited to only three studies. Likewise, there were insufficient studies to quantify the association between income and hospital admissions and mortality. The studies included in the meta-analysis predominantly defined exacerbations by ED visits, hospitalisations and/or OCS. The exacerbation rate ranges from <1% to 29% in the included studies, or <10 exacerbations per 1000 patients vs. 290 exacerbations per 1000 patients.^46^^,^^55^ Depending on the baseline exacerbation rate, the pooled OR estimate of 1.25 could pose an uneven burden on EDs. In practice, this could translate from as little as 500 more events per 100,000 patients per year (1–2 more events per day) to as high as 7200 per 100,000 patients per year (approximately 20 more per day), which could overwhelm EDs. Considering these findings mainly reflect severe exacerbations, the results are likely skewed, and the burden on secondary care will likely be overestimated. Nonetheless, more evidence is required to assess the associations between income and asthma outcomes beyond severe exacerbations and between unemployment and asthma outcomes.

The subgroup analysis showed no significant difference, with the variation in the definition of exacerbation explained mainly by the heterogeneity in adults. Further studies need to address the heterogeneity in adults. Also, only one moderator was examined as other possible effect-modifying study-related factors were subject to reporting differences in sex (by intervention or exposure), age (mean, median and frequency) and ethnic groups. Therefore, we cannot rule out the impact of these moderators on the associations.

**Fig. 4:**
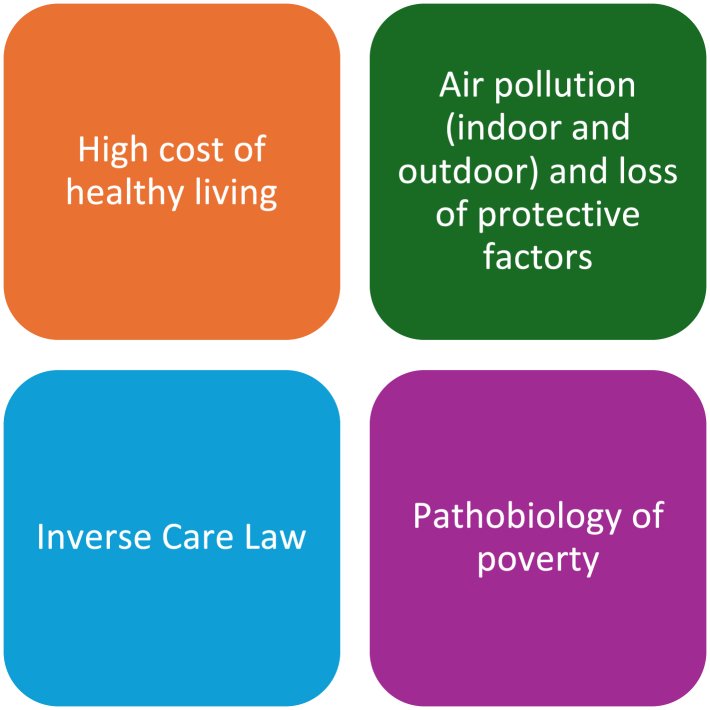
**A matrix illustrating the synergistic effects of material disadvantages on poor asthma outcomes**.

Our findings have research, clinical, and policy implications. Since all studies investigated the association at a high-income/employment level, there is scope to disentangle the mechanisms underpinning these inequalities in disadvantaged populations. Not only does this indicate a persistent issue that has yet to be resolved, but it is also multifaceted. Employment and income represent material disadvantages, i.e., limited wealth and access to goods and conveniences (Fig. 4).^109^ As living a healthy life is prohibitively expensive, the most deprived are more likely to live in substandard housing and have poor nutrition. These adverse exposures may work synergistically against the most deprived groups, who are excessively exposed to indoor and outdoor air pollution while lacking the protective effects of a healthy diet to stimulate an immune response.^110^^,^^111^ This can have pathobiological effects on people born into deprivation, who have worse airways or who are already born in a pro-inflammatory state.^112^^,^^113^ Evidence indicates that the incidence of developing asthma is statistically significantly increased when children are exposed early in life.^114^ The most deprived children were reported to have higher levels of airway inflammation and worse lung function.^115^ Meanwhile, these groups are affected by The Inverse Care Law: the “availability of good medical care tends to vary inversely with the need for it in the population served”.^116^ Given their precarious working arrangement, such as zero-hour contracts, leaving work to access appointments is expensive. Not only is there the risk of loss of income and employment but also the risk of long-term poorer health outcomes from restricted access to healthcare.^112^ These are likely to have knock-on effects on education, health, housing, and the wider environment.^117^ Improving the material disadvantages can improve the “structural (e.g., systemic racism); social (e.g., socioeconomic status [SES]); biological (e.g., genetics); and behavioural (e.g., smoking) factors” driving the widespread disparities.^118^ Addressing the material disadvantages is paramount to improving widespread disparities, which will require researchers to explore at the domain level rather than the aggregate level.

When developing interventions, clinicians need to understand how underlying mechanisms manifest in the clinical pathway and affect outcomes. Here, clinicians should consider the co-existing drivers for inequality that may have synergistic effects, where the Inverse Care Law may manifest in clinical pathways, and how the pathobiology of poverty might impact clinical outcomes (and what can be done to address this). This can support efforts to advance knowledge of the interactions between environmental, genetic and immunological factors, as well as policies highlighting the need for equity in reducing health disparities.^13^

Policymakers could reduce the effects of income and unemployment on asthma outcomes by improving housing conditions, especially for at-risk populations like children, and unemployment-induced stress. Despite the housing shortages and the need to build new homes, houses should be free from indoor and outdoor pollution. Offering a tailored package of housing improvements, such as remediation, to low-income households should include incentives such as shopping vouchers and credit to utility bills.^119^ Amid welfare reform, including changes to unemployment benefits, policymakers should provide further psychological resources and career support to facilitate government efforts to ‘get Britain working again’. Policies should embed mental health support in employment programmes, offering regular contact and health checks (including asthma reviews) until the individual finds a job.^120^ Should these policies be implemented successfully, more people will be working and earning an income. Living in a secure and stable environment means less disposable income is spent on repairs and maintenance. Addressing these widespread disparities will improve asthma outcomes and reduce overall inequalities in the long term.

In conclusion, income is an important indicator of SES that is associated with severe asthma exacerbations in children and adults. In contrast, we were unable to determine the role of unemployment in asthma outcomes. More evidence is required to assess the associations between income and asthma outcomes beyond severe exacerbations and unemployment and asthma outcomes more generally. Domain-level studies can help fully understand the mechanisms underlying these associations. Studies also need to investigate further the heterogeneity in adults. Interventions addressing the material disadvantages can inform and better target policies, such as improved housing conditions and unemployed-related stress. These will help researchers, clinicians, and policymakers get closer to improving asthma outcomes and reducing health inequalities.

## Contributors

ZG, AS, IS, GAD, HW, CK and JKQ conceived the study design. ZG, IDK, EM, AMA and AT screened the studies for inclusion and extracted the data. ZG assessed the studies for risk of bias, conducted the meta-analysis and drafted the manuscript. HW and CK provided methodological and statistical support. AS, IS, GAD, CK, HW and JKQ edited and reviewed the manuscript. HW, CK and JKQ provided supervision. ZG, HW, CK and JKQ accessed and verified the underlying data. All authors had full access to all the data in the study and had final responsibility for the decision to submit for publication.

## Data sharing statement

Requests for data can be made to the corresponding author.

## Declaration of interests

AS has received grant funding from Health Data Research UK (to his institution). JKQ has been supported by institutional research grants from the Medical Research Council, NIHR, Health Data Research, GSK, BI, AZ, Insmed, Sanofi and received personal fees for advisory board participation, consultancy or speaking fees from GlaxoSmithKline, BI, Sanofi, Chiesi, AstraZeneca. ZG, IDK, EM, AMA, AT, GAD, HW and CK declare no competing interests.
