# Supplementary material for: Association between income, employment status, and asthma outcomes: a systematic review and meta-analysis
Source: Lancet Reg Health Eur. 2025 Jun 26;56:101367. doi: 10.1016/j.lanepe.2025.101367 (PMC12268008; doi:10.1016/j.lanepe.2025.101367)
Supplement: Supplementary material Appendix S1 and Tables S1–S6 [file mmc1.docx]

**Supplementary material**

Table of Contents:

[Table S1: PRISMA Checklist 2](#_Toc197871413)

[Table S2: Search strategy (07/03/2024-03/04/2025) 5](#_Toc197871414)

[A. Search terms 5](#_Toc197871415)

[B. Embase 6](#_Toc197871416)

[C. MEDLINE 8](#_Toc197871417)

[Table S3: PICOS framework for the inclusion and exclusion criteria. 10](#_Toc197871418)

[Table S4: Full-text screening (excluded papers) 11](#_Toc197871419)

[Table S5: Risk of Bias 12](#_Toc197871420)

[A. Risk Of Bias In Non-randomized Studies - of Exposure (ROBINS-E) tool 12](#_Toc197871421)

[B. Adapted Risk of Bias framework for cross-sectional studies 14](#_Toc197871422)

[C. Revised Cochrane risk-of-bias tool for randomized trials (RoB 2) 15](#_Toc197871423)

[Table S6: Study outcomes, definitions and covariates 24](#_Toc197871424)

[A. Exacerbations 24](#_Toc197871425)

[B. Hospital admissions 25](#_Toc197871426)

[C. Mortality 26](#_Toc197871427)

[D. Covariates 27](#_Toc197871428)

[Appendix 1: Deriving odds ratio from Shor et al (2017)^30^ conversion formula 34](#_Toc197871429)

[References 35](#_Toc197871430)

## **Table S1: PRISMA Checklist**

| **Section and Topic** | **Item #** | **Checklist item** | **Location where item is reported #** |
| --- | --- | --- | --- |
| **TITLE** | | |  |
| Title | 1 | Identify the report as a systematic review. | 0 |
| **ABSTRACT** | | |  |
| Abstract | 2 | See the PRISMA 2020 for Abstracts checklist. | 1 |
| **INTRODUCTION** | | |  |
| Rationale | 3 | Describe the rationale for the review in the context of existing knowledge. | 3 |
| Objectives | 4 | Provide an explicit statement of the objective(s) or question(s) the review addresses. | 3 |
| **METHODS** | | |  |
| Eligibility criteria | 5 | Specify the inclusion and exclusion criteria for the review and how studies were grouped for the syntheses. | 4, Supplementary material table S3 (p.10) |
| Information sources | 6 | Specify all databases, registers, websites, organisations, reference lists and other sources searched or consulted to identify studies. Specify the date when each source was last searched or consulted. | 4 |
| Search strategy | 7 | Present the full search strategies for all databases, registers and websites, including any filters and limits used. | Supplementary material table S2 (pp.5-9) |
| Selection process | 8 | Specify the methods used to decide whether a study met the inclusion criteria of the review, including how many reviewers screened each record and each report retrieved, whether they worked independently, and if applicable, details of automation tools used in the process. | 4 |
| Data collection process | 9 | Specify the methods used to collect data from reports, including how many reviewers collected data from each report, whether they worked independently, any processes for obtaining or confirming data from study investigators, and if applicable, details of automation tools used in the process. | 4 |
| Data items | 10a | List and define all outcomes for which data were sought. Specify whether all results that were compatible with each outcome domain in each study were sought (e.g. for all measures, time points, analyses), and if not, the methods used to decide which results to collect. | 4, Supplementary material table S3 (p.10) |
|  | 10b | List and define all other variables for which data were sought (e.g. participant and intervention characteristics, funding sources). Describe any assumptions made about any missing or unclear information. | 4 |
| Study risk of bias assessment | 11 | Specify the methods used to assess risk of bias in the included studies, including details of the tool(s) used, how many reviewers assessed each study and whether they worked independently, and if applicable, details of automation tools used in the process. | 4 |
| Effect measures | 12 | Specify for each outcome the effect measure(s) (e.g. risk ratio, mean difference) used in the synthesis or presentation of results. | 4-5 |
| Synthesis methods | 13a | Describe the processes used to decide which studies were eligible for each synthesis (e.g. tabulating the study intervention characteristics and comparing against the planned groups for each synthesis (item #5)). | N/A |
|  | 13b | Describe any methods required to prepare the data for presentation or synthesis, such as handling of missing summary statistics, or data conversions. | 5, Supplementary material appendix 1 (p.34) |
|  | 13c | Describe any methods used to tabulate or visually display results of individual studies and syntheses. | 4-5 |
|  | 13d | Describe any methods used to synthesize results and provide a rationale for the choice(s). If meta-analysis was performed, describe the model(s), method(s) to identify the presence and extent of statistical heterogeneity, and software package(s) used. | 5 |
|  | 13e | Describe any methods used to explore possible causes of heterogeneity among study results (e.g. subgroup analysis, meta-regression). | 5 |
|  | 13f | Describe any sensitivity analyses conducted to assess robustness of the synthesized results. | N/A |
| Reporting bias assessment | 14 | Describe any methods used to assess risk of bias due to missing results in a synthesis (arising from reporting biases). | N/A |
| Certainty assessment | 15 | Describe any methods used to assess certainty (or confidence) in the body of evidence for an outcome. | N/A |
| **RESULTS** | | |  |
| Study selection | 16a | Describe the results of the search and selection process, from the number of records identified in the search to the number of studies included in the review, ideally using a flow diagram. | Figure 1 |
|  | 16b | Cite studies that might appear to meet the inclusion criteria, but which were excluded, and explain why they were excluded. | Supplementary material table s4 (p.11) |
| Study characteristics | 17 | Cite each included study and present its characteristics. | Table 1, Supplementary material table s6a-d (pp.24-33) |
| Risk of bias in studies | 18 | Present assessments of risk of bias for each included study. | Table 2, Supplementary material table s5a-c (pp.12-23) |
| Results of individual studies | 19 | For all outcomes, present, for each study: (a) summary statistics for each group (where appropriate) and (b) an effect estimate and its precision (e.g. confidence/credible interval), ideally using structured tables or plots. | Table 1, Supplementary material table s6a-c (pp.24-26) |
| Results of syntheses | 20a | For each synthesis, briefly summarise the characteristics and risk of bias among contributing studies. | 7, Table 3 |
|  | 20b | Present results of all statistical syntheses conducted. If meta-analysis was done, present for each the summary estimate and its precision (e.g. confidence/credible interval) and measures of statistical heterogeneity. If comparing groups, describe the direction of the effect. | 7, Figure 2-3 |
|  | 20c | Present results of all investigations of possible causes of heterogeneity among study results. | N/A |
|  | 20d | Present results of all sensitivity analyses conducted to assess the robustness of the synthesized results. | N/A |
| Reporting biases | 21 | Present assessments of risk of bias due to missing results (arising from reporting biases) for each synthesis assessed. | N/A |
| Certainty of evidence | 22 | Present assessments of certainty (or confidence) in the body of evidence for each outcome assessed. | N/A |
| **DISCUSSION** | | |  |
| Discussion | 23a | Provide a general interpretation of the results in the context of other evidence. | 8-9 |
|  | 23b | Discuss any limitations of the evidence included in the review. | 9-10 |
|  | 23c | Discuss any limitations of the review processes used. | 10 |
|  | 23d | Discuss implications of the results for practice, policy, and future research. | 10-11 |
| **OTHER INFORMATION** | | |  |
| Registration and protocol | 24a | Provide registration information for the review, including register name and registration number, or state that the review was not registered. | 3 |
|  | 24b | Indicate where the review protocol can be accessed, or state that a protocol was not prepared. | 3 |
|  | 24c | Describe and explain any amendments to information provided at registration or in the protocol. | N/A |
| Support | 25 | Describe sources of financial or non-financial support for the review, and the role of the funders or sponsors in the review. | 5,12 |
| Competing interests | 26 | Declare any competing interests of review authors. | 11,12 |
| Availability of data, code and other materials | 27 | Report which of the following are publicly available and where they can be found: template data collection forms; data extracted from included studies; data used for all analyses; analytic code; any other materials used in the review. | N/A |

*From:*  Page MJ, McKenzie JE, Bossuyt PM, Boutron I, Hoffmann TC, Mulrow CD, et al. The PRISMA 2020 statement: an updated guideline for reporting systematic reviews. BMJ 2021;372:n71. doi: 10.1136/bmj.n71

For more information, visit: <http://www.prisma-statement.org/>

## **Table S2: Search strategy (07/03/2024-03/04/2025)**

### **Search terms**

| **Databases** | **Search terms** |
| --- | --- |
| Embase &  MEDLINE | (income OR poverty OR disadvantage* OR depriv* OR wealth* OR $employ$ OR job* OR occupation* OR $economic)  AND  (asthma)  AND  (all-cause mortality OR exacerbation* OR $emergenc$ OR $admission$) |

### **Embase**

| **#** | **Query** | **Results from 7 March 2024** |
| --- | --- | --- |
| 1 | income/ | 73,408 |
| 2 | poor.mp. | 1,143,242 |
| 3 | poverty/ | 58,492 |
| 4 | disadvantage*.mp. | 131,864 |
| 5 | depriv*.mp. | 176,619 |
| 6 | wealth*.mp. | 34,978 |
| 7 | $employ$.mp. | 1,066,795 |
| 8 | job*.mp. | 159,908 |
| 9 | occupation*.mp. | 461,930 |
| 10 | $economic.mp. | 527,322 |
| 11 | work/ | 40,136 |
| 12 | 1 or 2 or 3 or 4 or 5 or 6 or 7 or 8 or 9 or 10 or 11 | 3,474,178 |
| 13 | asthma/ | 294,895 |
| 14 | all cause mortality/ or mortality/ | 1,043,684 |
| 15 | exacerbation*.mp. | 264,541 |
| 16 | $emergency.mp. | 727,233 |
| 17 | $admission$.mp. | 683,718 |
| 18 | 14 or 15 or 16 or 17 | 241,5604 |
| 19 | 12 and 13 and 18 | **7,429** |
| 20 | limit 19 to (human and english language) | **6,806** |

| **#** | **Query** | **Results from 15 March 2024** |
| --- | --- | --- |
| 1 | income.mp | 287,560 |
| 2 | poverty.mp | 75,333 |
| 3 | disadvantage*.mp. | 132,107 |
| 4 | depriv*.mp. | 176,828 |
| 5 | wealth*.mp. | 35,040 |
| 6 | $employ$.mp. | 1,069,286 |
| 7 | job*.mp. | 160,125 |
| 8 | occupation*.mp. | 462,335 |
| 9 | $economic.mp. | 528,293 |
| 10 | work.mp | 1,883,774 |
| 11 | 1 or 2 or 3 or 4 or 5 or 6 or 7 or 8 or 9 or 10 | 4,124,468 |
| 12 | asthma.mp | 365,982 |
| 13 | all-cause mortality.ab,ti. | 87,629 |
| 14 | exacerbation*.ab,ti. | 117,436 |
| 15 | $emergenc$.ab,ti. | 725,995 |
| 16 | $admission$.ab,ti. | 533,826 |
| 17 | 13 or 14 or 15 or 16 | 1,356,774 |
| 18 | 11 and 12 and 17 | **6,104** |
| 19 | limit 18 to (human and english language and yr="2010 -Current") | **4,430** |

| **#** | **Query** | **Results from 18 March 2024** |
| --- | --- | --- |
| 1 | income.ab,ti. | 229,952 |
| 2 | poverty.ab,ti. | 40,928 |
| 3 | disadvantage*.ab,ti. | 131,536 |
| 4 | depriv*.ab,ti. | 145,551 |
| 5 | wealth*.ab,ti. | 34,844 |
| 6 | $employ$.ab,ti. | 1,019,989 |
| 7 | job*.ab,ti. | 105,146 |
| 8 | occupation*.ab,ti. | 256,624 |
| 9 | $economic.ab,ti. | 388,098 |
| 10 | 1 or 2 or 3 or 4 or 5 or 6 or 7 or 8 or 9 | 2,119,388 |
| 11 | asthma.ab,ti. | 266,011 |
| 12 | all-cause mortality.ab,ti. | 87,816 |
| 13 | exacerbation*.ab,ti. | 117,543 |
| 14 | $emergenc$.ab,ti. | 727,191 |
| 15 | $admission$.ab,ti. | 534,580 |
| 16 | 12 or 13 or 14 or 15 | 1,358,829 |
| 17 | 10 and 11 and 16 | **3,603** |
| 18 | limit 17 to (human and english language and yr="2010 -Current") | **2,583** |

| **#** | **Query** | **Results from 3 April 2025** |
| --- | --- | --- |
| 1 | income.ab,ti. | 253,230 |
| 2 | poverty.ab,ti. | 43,995 |
| 3 | disadvantage*.ab,ti. | 139,283 |
| 4 | depriv*.ab,ti. | 154,636 |
| 5 | wealth*.ab,ti. | 37,574 |
| 6 | $employ$.ab,ti. | 1,143,763 |
| 7 | job*.ab,ti. | 113,370 |
| 8 | occupation*.ab,ti. | 271,595 |
| 9 | $economic.ab,ti. | 420,206 |
| 10 | 1 or 2 or 3 or 4 or 5 or 6 or 7 or 8 or 9 | 2,321,468 |
| 11 | asthma.ab,ti. | 280,558 |
| 12 | all-cause mortality.ab,ti. | 99,351 |
| 13 | exacerbation*.ab,ti. | 125,648 |
| 14 | $emergenc$.ab,ti. | 784,986 |
| 15 | $admission$.ab,ti. | 572,515 |
| 16 | 12 or 13 or 14 or 15 | 1,465,024 |
| 17 | 10 and 11 and 16 | **3,882** |
| 18 | limit 17 to (human and english language and yr="2010 -Current") | **2,872** |

### **MEDLINE**

| **#** | **Query** | **Results from 7 March 2024** |
| --- | --- | --- |
| 1 | Income/ | 35,495 |
| 2 | poor.mp. | 777,258 |
| 3 | Poverty/ | 44,426 |
| 4 | disadvantage*.mp. | 100,914 |
| 5 | depriv*.mp. | 127,872 |
| 6 | wealth*.mp. | 30,789 |
| 7 | $employ$.mp. | 847,172 |
| 8 | job*.mp. | 112,667 |
| 9 | occupation*.mp. | 372,964 |
| 10 | $economic.mp. | 346,373 |
| 11 | Work/ | 20,250 |
| 12 | 1 or 2 or 3 or 4 or 5 or 6 or 7 or 8 or 9 or 10 or 11 | 2,540,035 |
| 13 | Asthma/ | 141,802 |
| 14 | Mortality/ | 49,752 |
| 15 | exacerbation*.mp. | 66,851 |
| 16 | $emergency.mp. or Emergencies/ | 431,381 |
| 17 | $admission$.mp. or Hospitalisation/ | 409,124 |
| 18 | 14 or 15 or 16 or 17 | 887,543 |
| 19 | 12 and 13 and 18 | **2,386** |
| 20 | limit 19 to (english language and humans) | **2,227** |

| **#** | **Query** | **Results from 15 March 2024** |
| --- | --- | --- |
| 1 | income.mp | 205,746 |
| 2 | poverty.mp | 74,125 |
| 3 | disadvantage*.mp. | 101,055 |
| 4 | depriv*.mp. | 127,994 |
| 5 | wealth*.mp. | 30,852 |
| 6 | $employ$.mp. | 849,199 |
| 7 | job*.mp. | 112,823 |
| 8 | occupation*.mp. | 373,217 |
| 9 | $economic.mp. | 347,069 |
| 10 | work.mp | 1,425,905 |
| 11 | 1 or 2 or 3 or 4 or 5 or 6 or 7 or 8 or 9 or 10 | 3,139,078 |
| 12 | asthma.mp | 203,012 |
| 13 | all-cause mortality.ab,ti. | 55,446 |
| 14 | exacerbation*.ab,ti. | 66,389 |
| 15 | $emergenc$.ab,ti. | 515,659 |
| 16 | $admission$.ab,ti. | 297,713 |
| 17 | 13 or 14 or 15 or 16 | 877,697 |
| 18 | 11 and 12 and 17 | **2,941** |
| 19 | limit 18 to (human and english language and yr="2010 -Current") | **1,439** |

| **#** | **Query** | **Results from 18 March 2024** |
| --- | --- | --- |
| 1 | income.ab,ti. | 184,803 |
| 2 | poverty.ab,ti. | 34,370 |
| 3 | disadvantage*.ab,ti. | 100,788 |
| 4 | depriv*.ab,ti. | 107,385 |
| 5 | wealth*.ab,ti. | 30,772 |
| 6 | $employ$.ab,ti. | 807,107 |
| 7 | job*.ab,ti. | 82,937 |
| 8 | occupation*.ab,ti. | 189,510 |
| 9 | $economic.ab,ti. | 308,041 |
| 10 | 1 or 2 or 3 or 4 or 5 or 6 or 7 or 8 or 9 | 1,657,965 |
| 11 | asthma.ab,ti. | 171,165 |
| 12 | all-cause mortality.ab,ti. | 55,514 |
| 13 | exacerbation*.ab,ti. | 66,413 |
| 14 | $emergenc$.ab,ti. | 515,986 |
| 15 | $admission$.ab,ti. | 297,882 |
| 16 | 12 or 13 or 14 or 15 | 878,244 |
| 17 | 10 and 11 and 16 | **2,046** |
| 18 | limit 17 to (human and english language and yr="2010 -Current") | **1,049** |

| **#** | **Query** | **Results from 3 April 2025** |
| --- | --- | --- |
| 1 | income.ab,ti. | 204,466 |
| 2 | poverty.ab,ti. | 37,052 |
| 3 | disadvantage*.ab,ti. | 107,479 |
| 4 | depriv*.ab,ti. | 113,607 |
| 5 | wealth*.ab,ti. | 33,424 |
| 6 | $employ$.ab,ti. | 918,920 |
| 7 | job*.ab,ti. | 88,658 |
| 8 | occupation*.ab,ti. | 199,703 |
| 9 | $economic.ab,ti. | 337,298 |
| 10 | 1 or 2 or 3 or 4 or 5 or 6 or 7 or 8 or 9 | 1,833,604 |
| 11 | asthma.ab,ti. | 178,305 |
| 12 | all-cause mortality.ab,ti. | 63,139 |
| 13 | exacerbation*.ab,ti. | 71,084 |
| 14 | $emergenc$.ab,ti. | 557,429 |
| 15 | $admission$.ab,ti. | 318,532 |
| 16 | 12 or 13 or 14 or 15 | 947,768 |
| 17 | 10 and 11 and 16 | **2,207** |
| 18 | limit 17 to (human and english language and yr="2010 -Current") | **1,478** |

## **Table S3: PICOS framework for the inclusion and exclusion criteria.**

| **Criteria** | **Included** | **Excluded** |
| --- | --- | --- |
| **Population** | Asthma-diagnosed patients determined through primary or secondary care (clinically diagnosed or self-reported) | Non-asthma patients |
| **Intervention** | Income level (high vs low income) or  employment status (employed or unemployed) or socioeconomic status/index (highest vs lowest) including self-reported | Non-income, employment or socioeconomic measures or comparator. |
| **Comparator** | 1. Income level (high vs low income) based on gross domestic product, gross national income or purchasing power parity. These measures can be in any currency and type, i.e., real (inflation-adjusted), gross, or net (disposable/household) applied to the whole population or per capita 2. Employment status (employed/unemployed) | Non-income, employment or socioeconomic measures or comparator |
| **Outcomes** | 1. Exacerbations based on primary and secondary care definitions of mild and severe exacerbations 2. Hospital admissions (all, non-emergency and emergency) 3. Mortality, including all-cause and asthma-related death | Non-asthma-related outcomes, asthma management, or process-based measures |
| **Study** | Quantitative (any reported data that describes or quantifies the association (adjusted or unadjusted) | Qualitative (unreported data, reviews, opinion pieces, reports and unpublished grey literature) |

## **Table S4: Full-text screening (excluded papers)**

| **Paper** | **Justification** |
| --- | --- |
| Ungar et al (2010)^1^ | Abstract |
| Heffernan et al (2011)^2^ | Abstract |
| Tamulis & Krupitsky (2011)^3^ | Abstract |
| Grecian et al (2013)^4^ | Abstract |
| Von Bülow et al (2015)^5^ | Abstract |
| Slain et al (2016)^6^ | Abstract |
| Yakubovich et al (2016)^7^ | Abstract |
| Al Sallakh et al (2017)^8^ | Abstract |
| Chang et al (2021)^9^ | Abstract |
| Håkansson et al (2022)^10^ | Abstract |
| Khamooshi et al (2022)^11^ | Abstract |
| Skeen et al (2023)^12^ | Abstract |
| Hussain et al (2023)^13^ | Abstract |
| Francis-Morel et al (2023)^14^ | Abstract |
| Padasak (2024)^15^ | Abstract |
| Alsallakh et al (2023)^16^ | Book chapter |
| Schlichting et al (2021)^17^ | Unreported data |
| Crighton et al (2010)^18^ | Self-reported asthma patients |
| Vowles et al (2020)^19^ | Self-reported asthma patients and exposure is not of interest |
| Moncrief et al (2014)^20^ | Exposure is not of interest (single parent households) |
| Sakai-Bizmark et al (2019)^21^ | Exposure is not of interest (homelessness) and not comparable (not in terms of low vs high) |
| De La Cruz et al (2021)^22^ | Exposure is not of interest (insurance) |
| Grant et al (2025)^23^ | Exposure is not of interest (housing mobility) |
| Gaffney et al (2024)^24^ | Exposure is not of interest (SES not mentioned when describing age trajectory of asthma hospitalisation) |
| Lim et al (2024)^25^ | Outcome is not of interest (not asthma-related per se) |
| Thakur et al (2014)^26^ | Outcome (asthma control) is not of interest |
| Kim et al (2018)^27^ | Population (non-asthma residents) and outcome is not of interest (incident asthma diagnosis) |
| Antunes et al (2013)^28^ | Not in the English language |
| Cardet et al (2016)^29^ | Duplicate (different year, same publication) |

## **Table S5: Risk of Bias**

### **Risk Of Bias In Non-randomized Studies - of Exposure (ROBINS-E) tool**

| **Bias domain** | **Ungar et al. (2011)** | **Auger et al. (2013)** | **To et al. (2014)** | **Zhang et al. (2017)** | **Grunwell et al. (2018)** | **Mazalovic et al. (2018)** | **Seibert et al. (2019)** | **Brite et al. (2020)** | **Jroundi & Tse (2021)** | **Alsallakh et al. (2021)** | **Busby et al. (2021)** | **Kallis et al. (2023)** |
| --- | --- | --- | --- | --- | --- | --- | --- | --- | --- | --- | --- | --- |
| **Preliminary considerations**  Confounders  (if **☹**, high risk of bias) | **☺** | **☺** | **☺** | **☺** | **☺** | **☹** | **☺** | **☺** | **☺** | **☺** | **☺** | **☺** |
| **Domain 1:** Risk of bias due to confounding | **😐** | **😐** | **☺** | **☹** | **☺** |  | **☺** | **☺** | **☺** | **😐** | **☺** | **☺** |
| **Domain 2:** Risk of bias arising from measurement of the exposure | **☺** | **☺** | **☺** | **☺** | **☺** |  | **☺** | **☺** | **☺** | **☺** | **☺** | **☺** |
| **Domain 3:** Risk of bias in selection of participants into the study (or into the analysis) | **☺** | **☺** | **☺** | **☺** | **☺** |  | **☺** | **☺** | **☺** | **☺** | **☺** | **☺** |
| **Domain 4:** Risk of bias due to post-exposure interventions | **☺** | **☺** | **☺** | **☺** | **☺** |  | **☺** | **☺** | **☺** | **☺** | **☺** | **☺** |
| **Domain 5:** Risk of bias due to missing data | **☺** | **☺** | **☺** | **☺** | **☺** |  | **☺** | **☺** | **☺** | **☺** | **☺** | **☺** |
| **Domain 6:** Risk of bias arising from measurement of the outcome | **☺** | **☺** | **☺** | **☺** | **☺** |  | **☺** | **☺** | **☺** | **☺** | **☺** | **☺** |
| **Domain 7:** Risk of bias in selection of the reported result | **☹** | **☺** | **☺** | **☺** | **☺** |  | **☺** | **☺** | **☺** | **☺** | **☺** | **☺** |
| **Overall risk of bias** | **😐** | **☺** | **☺** | **☹** | **☺** | **☹** | **☺** | **☺** | **☺** | **☺** | **☺** | **☺** |

**Key:**

- Green smiley face = low risk
- Yellow straight face = moderate or unclear risk
- Red sad face = high risk

**NB:** The prespecified confounders for preliminary consideration are at least one of age, sex and ethnicity. All confounders are not specific to any exposure-outcome combinations; all apply to the outcome. However, they can also be moderators or mediators.

| **Bias domain** | **Khalaf et al. (2024)** | **Renzi-Lomholt et al. (2024)** | **Simms-Wiliams et al. (2024)** | **Gaietto et al. (2024)** | **Khalid et al. (2024)** | **Xu et al. (2024)** | **Miller et al. (2025)** |
| --- | --- | --- | --- | --- | --- | --- | --- |
| **Preliminary considerations**  Confounders  (if **☹**, high risk of bias) | **☺** | **☺** | **☺** | **☺** | **☺** | **☺** | **☺** |
| **Domain 1:** Risk of bias due to confounding | **☺** | **☺** | **☺** | **☺** | **☺** | **😐** | **☺** |
| **Domain 2:** Risk of bias arising from measurement of the exposure | **☺** | **☺** | **☺** | **☺** | **☺** | **☺** | **😐** |
| **Domain 3:** Risk of bias in selection of participants into the study (or into the analysis) | **☺** | **☺** | **☺** | **😐** | **☺** | **☺** | **😐** |
| **Domain 4:** Risk of bias due to post-exposure interventions | **☺** | **☺** | **☺** | **☺** | **☺** | **☺** | **☺** |
| **Domain 5:** Risk of bias due to missing data | **☹** | **☺** | **☺** | **☺** | **☺** | **☺** | **☺** |
| **Domain 6:** Risk of bias arising from measurement of the outcome | **☺** | **☺** | **☺** | **☺** | **☺** | **☺** | **☺** |
| **Domain 7:** Risk of bias in selection of the reported result | **☺** | **☺** | **☺** | **☺** | **☺** | **☺** | **☺** |
| **Overall risk of bias** | **😐** | **☺** | **☺** | **☺** | **☺** | **☺** | **😐** |

**Key:**

- Green smiley face = low risk
- Yellow straight face = moderate or unclear risk
- Red sad face = high risk

**NB:** The prespecified confounders for preliminary consideration are at least one of age, sex and ethnicity. All confounders are not specific to any exposure-outcome combinations; all apply to the outcome. However, they can also be moderators or mediators.

### **Adapted Risk of Bias framework for cross-sectional studies**

| **Bias domain** | **Disano et al. (2010)** | **Law et al. (2011)** | **Gupta et al. (2018)** | **Eum et al. (2019)** | **Molina et al. (2019)** | **Mukherjee et al. (2022)** | **Cardet et al. (2022)** | **Akinyemi et al. (2024)** | **Scott et al. (2024)** | **Skeen et al. (2024)** |
| --- | --- | --- | --- | --- | --- | --- | --- | --- | --- | --- |
| **Selection**  (appropriateness of eligibility criteria; comparability of exposure group vs comparison group; nonresponse rate; nonresponse rate – mechanism; recruitment time frame; representativeness of sample to target population) | **☺** | **☺** | **☺** | **☺** | **😐** | **☺** | **😐** | **☺** | **☹** | **☺** |
| **Exposure**  (validity and reliability of exposure measurement) | **☺** | **☺** | **☺** | **☺** | **☺** | **☺** | **☺** | **☺** | **😐** | **☺** |
| **Outcome**  (blinding of the research staff; validity and reliability of outcome measurement) | **☺** | **☺** | **☺** | **☺** | **☺** | **☺** | **☺** | **☺** | **😐** | **☺** |
| **Confounding**  (amount for confounding variables; description of confounding variables) | **☹** | **☺** | **😐** | **😐** | **☺** | **😐** | **☺** | **☺** | **😐** | **☺** |
| **Missingness**  (amount of missingness; handling of missingness; mechanism of missingness) | **😐** | **😐** | **☺** | **😐** | **☺** | **☺** | **😐** | **😐** | **😐** | **☺** |
| **Selective reporting**  (selective reporting of outcomes) | **☺** | **☺** | **☺** | **☺** | **☺** | **☺** | **☺** | **😐** | **☺** | **☺** |
| **Conflict of interest**  (funding) | **☺** | **☺** | **☺** | **☺** | **☺** | **☺** | **☺** | **😐** | **☺** | **☺** |
| **Other bias**  (other bias or threats to internal validity) | **☹** | **😐** | **☺** | **☺** | **☺** | **☺** | **☺** | **☺** | **☹** | **☺** |
| **Overall risk of bias** | **☹** | **☺** | **☺** | **☺** | **☺** | **☺** | **😐** | **😐** | **☹** | **☺** |

**Key:**

- Green smiley face = low risk
- Yellow straight face = moderate or unclear risk
- Red sad face = high risk

### **Revised Cochrane risk-of-bias tool for randomized trials (RoB 2)**

Edited by Julian PT Higgins, Jelena Savović, Matthew J Page, Jonathan AC Sterne
on behalf of the RoB2 Development Group

**Version of 22 August 2019**

#### **Cardet et al. (2018)**

The development of the RoB 2 tool was supported by the MRC Network of Hubs for Trials Methodology Research (MR/L004933/2- N61), with the support of the host MRC ConDuCT-II Hub (Collaboration and innovation for Difficult and Complex randomised controlled Trials In Invasive procedures - MR/K025643/1), by MRC research grant MR/M025209/1, and by a grant from The Cochrane Collaboration.


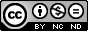


This work is licensed under a [Creative Commons Attribution-NonCommercial-NoDerivatives 4.0 International License](http://creativecommons.org/licenses/by-nc-nd/4.0/).

| **Study details**   \| **Reference** \| Cardet, J.C., Louisias, M., King, T.S., Castro, M., Codispoti, C.D., Dunn, R., Engle, L., Giles, B.L., Holguin, F., Lima, J.J., Long, D., Lugogo, N., Nyenhuis, S., Ortega, V.E., Ramratnam, S., Wechsler, M.E., Israel, E. & Phipatanakul, W. (2018) Income is an independent risk factor for worse asthma outcomes. *The Journal of allergy and clinical immunology*. 141 (2), 754-760.e3. doi:10.1016/j.jaci.2017.04.036. \| \| --- \| --- \|   **Study design**   \| X \| Individually-randomized parallel-group trial \| \| --- \| --- \| \| □ \| Cluster-randomized parallel-group trial \| \| □ \| Individually randomized cross-over (or other matched) trial \|   **For the purposes of this assessment, the interventions being compared are defined as**   \| Experimental: \| Vitamin D supplementation \| Comparator: \| Placebo \| \| --- \| --- \| --- \| --- \|  \| **Specify which outcome is being assessed for risk of bias** \| Secondary outcome: asthma exacerbations requiring systemic corticosteriods \| \| --- \| --- \|  \| **Specify the numerical result being assessed.** In case of multiple alternative analyses being presented, specify the numeric result (e.g. RR = 1.52 (95% CI 0.83 to 2.77) and/or a reference (e.g. to a table, figure or paragraph) that uniquely defines the result being assessed. \| Low v high income group and exacerbations  Poisson Regression Rate Ratio (IRR): 1.8 (1.1–3.1) \| \| --- \| --- \|   **Is the review team’s aim for this result…?**   \| □ \| to assess the effect of *assignment to intervention* (the ‘intention-to-treat’ effect) \| \| --- \| --- \| \| □ \| to assess the effect of *adhering to intervention* (the ‘per-protocol’ effect) \|   **If the aim is to assess the effect of *adhering to intervention***, select the deviations from intended intervention that should be addressed (at least one must be checked):  □ occurrence of non-protocol interventions  □ failures in implementing the intervention that could have affected the outcome  □ non-adherence to their assigned intervention by trial participants  **Which of the following sources were obtained to help inform the risk-of-bias assessment? (tick as many as apply)**  □ Journal article(s) with results of the trial  □ Trial protocol  □ Statistical analysis plan (SAP)  □ Non-commercial trial registry record (e.g. ClinicalTrials.gov record)  □ Company-owned trial registry record (e.g. GSK Clinical Study Register record)  □ “Grey literature” (e.g. unpublished thesis)  □ Conference abstract(s) about the trial  □ Regulatory document (e.g. Clinical Study Report, Drug Approval Package)  □ Research ethics application  □ Grant database summary (e.g. NIH RePORTER or Research Councils UK Gateway to Research)  □ Personal communication with trialist  □ Personal communication with the sponsor |
| --- | --- | --- | --- | --- | --- | --- | --- | --- | --- | --- | --- | --- | --- | --- | --- | --- | --- | --- | --- | --- |

**Risk of bias assessment**

Responses underlined in green are potential markers for low risk of bias, and responses in red are potential markers for a risk of bias. Where questions relate only to sign posts to other questions, no formatting is used.

**Domain 1: Risk of bias arising from the randomization process**

| **Signalling questions** | **Comments** | **Response options** |
| --- | --- | --- |
| **1.1 Was the allocation sequence random?** | “408 participants were randomized to placebo or cholecalciferol for 28 weeks as add-on therapy in the setting of a tapering ICS regimen” | Y / PY / PN / N / NI |
| **1.2 Was the allocation sequence concealed until participants were enrolled and assigned to interventions?** |  | Y / PY / PN / N / NI |
| **1.3 Did baseline differences between intervention groups suggest a problem with the randomization process?** |  | Y / PY / PN / N / NI |
| **Risk-of-bias judgement** |  | Low / High / Some concerns |
| Optional: What is the predicted direction of bias arising from the randomization process? |  | NA / Favours experimental / Favours comparator / Towards null /Away from null / Unpredictable |

Domain 2: Risk of bias due to deviations from the intended interventions (*effect of assignment to intervention*)

| **Signalling questions** | **Comments** | **Response options** |
| --- | --- | --- |
| **2.1. Were participants aware of their assigned intervention during the trial?** |  | Y / PY / PN / N / NI |
| **2.2. Were carers and people delivering the interventions aware of participants' assigned intervention during the trial?** |  | Y / PY / PN / N / NI |
| **2.3. If Y/PY/NI to 2.1 or 2.2: Were there deviations from the intended intervention that arose because of the trial context?** |  | NA / Y / PY / PN / N / NI |
| **2.4 If Y/PY to 2.3: Were these deviations likely to have affected the outcome?** |  | NA / Y / PY / PN / N / NI |
| **2.5. If Y/PY/NI to 2.4: Were these deviations from intended intervention balanced between groups?** |  | NA / Y / PY / PN / N / NI |
| **2.6 Was an appropriate analysis used to estimate the effect of assignment to intervention?** |  | Y / PY / PN / N / NI |
| **2.7 If N/PN/NI to 2.6: Was there potential for a substantial impact (on the result) of the failure to analyse participants in the group to which they were randomized?** |  | NA / Y / PY / PN / N / NI |
| **Risk-of-bias judgement** |  | Low / High / Some concerns |
| Optional: What is the predicted direction of bias due to deviations from intended interventions? |  | NA / Favours experimental / Favours comparator / Towards null /Away from null / Unpredictable |

Domain 2: Risk of bias due to deviations from the intended interventions (*effect of adhering to intervention*)

| **Signalling questions** | **Comments** | **Response options** |
| --- | --- | --- |
| **2.1. Were participants aware of their assigned intervention during the trial?** | n/a | Y / PY / PN / N / NI |
| **2.2. Were carers and people delivering the interventions aware of participants' assigned intervention during the trial?** |  | Y / PY / PN / N / NI |
| **2.3. [If applicable:] If Y/PY/NI to 2.1 or 2.2: Were important non-protocol interventions balanced across intervention groups?** | n/a | NA / Y / PY / PN / N / NI |
| **2.4. [If applicable:] Were there failures in implementing the intervention that could have affected the outcome?** | n/a | NA / Y / PY / PN / N / NI |
| **2.5. [If applicable:] Was there non-adherence to the assigned intervention regimen that could have affected participants’ outcomes?** | n/a | NA / Y / PY / PN / N / NI |
| **2.6. If N/PN/NI to 2.3, or Y/PY/NI to 2.4 or 2.5: Was an appropriate analysis used to estimate the effect of adhering to the intervention?** | n/a | NA / Y / PY / PN / N / NI |
| **Risk-of-bias judgement** | n/a | Low / High / Some concerns |
| Optional: What is the predicted direction of bias due to deviations from intended interventions? | n/a | NA / Favours experimental / Favours comparator / Towards null /Away from null / Unpredictable |

Domain 3: Missing outcome data

| **Signalling questions** | **Comments** | **Response options** |
| --- | --- | --- |
| **3.1 Were data for this outcome available for all, or nearly all, participants randomized?** |  | Y / PY / PN / N / NI |
| **3.2 If N/PN/NI to 3.1: Is there evidence that the result was not biased by missing outcome data?** |  | NA / Y / PY / PN / N |
| **3.3 If N/PN to 3.2: Could missingness in the outcome depend on its true value?** |  | NA / Y / PY / PN / N / NI |
| **3.4 If Y/PY/NI to 3.3: Is it likely that missingness in the outcome depended on its true value?** |  | NA / Y / PY / PN / N / NI |
| **Risk-of-bias judgement** |  | Low / High / Some concerns |
| Optional: What is the predicted direction of bias due to missing outcome data? | There was no mention of missing outcome data in the paper | NA / Favours experimental / Favours comparator / Towards null /Away from null / Unpredictable |

Domain 4: Risk of bias in measurement of the outcome

| **Signalling questions** | **Comments** | **Response options** |
| --- | --- | --- |
| **4.1 Was the method of measuring the outcome inappropriate?** | Appropriately measured based on a pre-specified treatment failure criteria and one or more of the following: failure to respond to rescue algorithm within 48 hours; FEV1 ≤50% of baseline or <40% of predicted (2 consecutive measurements); levalbuterol use of ≥16 puffs/day for 48 hours; exacerbation per physician opinion, and systemic corticosteroid treatment for asthma. | Y / PY / PN / N / NI |
| **4.2 Could measurement or ascertainment of the outcome have differed between intervention groups?** |  | Y / PY / PN / N / NI |
| **4.3 If N/PN/NI to 4.1 and 4.2: Were outcome assessors aware of the intervention received by study participants?** |  | NA / Y / PY / PN / N / NI |
| **4.4 If Y/PY/NI to 4.3: Could assessment of the outcome have been influenced by knowledge of intervention received?** |  | NA / Y / PY / PN / N / NI |
| **4.5 If Y/PY/NI to 4.4:** **Is it likely that assessment of the outcome was influenced by knowledge of intervention received?** |  | NA / Y / PY / PN / N / NI |
| **Risk-of-bias judgement** |  | Low / High / Some concerns |
| Optional: What is the predicted direction of bias in measurement of the outcome? |  | NA / Favours experimental / Favours comparator / Towards null /Away from null / Unpredictable |

Domain 5: Risk of bias in selection of the reported result

| **Signalling questions** | **Comments** | **Response options** |
| --- | --- | --- |
| **5.1 Were the data that produced this result analysed in accordance with a pre-specified analysis plan that was finalized before unblinded outcome data were available for analysis?** | “A detailed description of the recruitment, design, study visit structure and procedures including spirometry, and statistical analysis for the VIDA trial has been reported previously”  VIDA trial is a multicenter randomized double-blinded placebo-controlled trial | Y / PY / PN / N / NI |
| **Is the numerical result being assessed likely to have been selected, on the basis of the results, from...** |  |  |
| **5.2. ... multiple eligible outcome measurements (e.g. scales, definitions, time points) within the outcome domain?** | Outcome was prespecified and did not change during the course of the study. | Y / PY / PN / N / NI |
| **5.3 ... multiple eligible analyses of the data?** | Very unlikely as multiple analysis of secondary outcome (crude + adjusted) showed similar results and were reported | Y / PY / PN / N / NI |
| **Risk-of-bias judgement** |  | Low / High / Some concerns |
| Optional: What is the predicted direction of bias due to selection of the reported result? |  | NA / Favours experimental / Favours comparator / Towards null /Away from null / Unpredictable |

Overall risk of bias

| **Risk-of-bias judgement** |  | Low / High / Some concerns |
| --- | --- | --- |
| Optional: What is the overall predicted direction of bias for this outcome? |  | NA / Favours experimental / Favours comparator / Towards null /Away from null / Unpredictable |


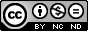


This work is licensed under a [Creative Commons Attribution-NonCommercial-NoDerivatives 4.0 International License](http://creativecommons.org/licenses/by-nc-nd/4.0/).

## **Table S6: Study outcomes, definitions and covariates**

### **Exacerbations**

| **Outcome** | **Paper** | **Cases (n)** | **Sample size (N)** | **Effect measure and size(s) (2dp)** |
| --- | --- | --- | --- | --- |
| Exacerbation | Law et al. (2011) | 2367 | 238,678 | OR=1·32 (1·03–1·68) |
|  | Ungar et al. (2011) | N/A | 490 | β=-0·33 (-0·64/-0·014) |
|  | Auger et al. (2013) | 135 | 601 | HR=1·82 (0·78–4·23) |
|  | Grunwell et al. (2018) | 170 | 579 | OR=1·28 (1·02–1·61) |
|  | Cardet et al. (2018) | N/A | 381 | IRR=1·80 (1·10–3·10) |
|  | Mazalovic et al. (2018) | 84 | 255 | OR=0·27 (0·09–0·84) |
|  | Seibert et al. (2019) | N/A | 342 | OR=0·94 (0·82–1·08) |
|  | Molina et al. (2020) | 149 | 664 | OR=1·09 (0·73–1·63) |
|  | Busby et al. (2021) | 5732 | 127,040 | OR=1·27 (1·13–1·42) |
|  | Jroundi & Tse (2021) | 9377 | 66,835 | HR=1·33 (1·15–1·53) |
|  | Cardet et al. (2022) | 596 | 990 | β=0·24 (0·11–0·38) |
|  | Kallis et al. (2023) | 93,625 | 805,138 | OR=1·06 (1·04–1·09) |
|  | Renzi-Lomholt et al. (2024) | 2353 | 29,851 | OR=0·68 (0·58–0·79) |
|  | Khalaf et al. (2024) | 5308 | 119,611 | Age 5-11·9 years: HR=1·2 (1·2–1·3)  Age 12-15·9 years: HR=1·4 (1·2–1·5)  Adolescents: HR=1.3 (1·2–1·5) |
|  | Gaietto et al. (2024) | 31 | 209 | OR=12·25 (2·59–57·97) |
|  | Skeen et al. (2024) | 142 | 193 | COI overall: OR=1·19 (0·92–1·54)  Household income ($5000 increments): OR=1·00 (0·89–1·12) |
|  | Xu et al. (2024) | 12,906 | 198,873 | RR=1·27 (1·20–1·35) |
|  | Scott et al. (2024) | 61 | 211 | Occupation: OR=0·690 (0·23–2·05)  Perceived financial status: OR=0·81 (0·27–2·50) |
|  | Miller et al. (2025) | 692 | 15,877 | Overall COI: IRR=1.26 (0·99–1·59)  Social and economic domain categories of COI: IRR=1·22 (0·97–1·53) |

### **Hospital admissions**

| **Outcome** | **Paper** | **Cases (n)** | **Sample size (N)** | **Effect measure and size(s) (2dp)** |
| --- | --- | --- | --- | --- |
| Admissions | Disano et al. (2010) | N/A | 46,173 urban areas | Age-standardised rates for low SES: 270 per 100,000  Age-standardised rates average SES: 196 per 100,000  Age-standardised rates high SES: 161 per 100,000 |
|  | Gupta et al. (2018) | 542,877 | N/A | 5-44: IRR=3·34 (3·30–3·38)  45-74: IRR=2·01 (1·98–2·05)  75+: IRR=1·43 (1·39–1·47) |
|  | Alsallakh et al. (2021) | ~3850 (derived from 3.6%) | 106,926 | IRR=1·56 (1·39–1·76) |
|  | Mukherjee et al. (2022) | N/A | 2110 | F=37·2, p<0·001  (One-way ANOVA) |
|  | Simms-Wiliams et al. (2024) | 17,506 | 1,385,326 | Asthma-related hospital admissions  5-11 years: IRR=1·51 (1·30–1·75)  2-17 years: IRR=1·52 (1·22–3·34)  8+ years: IRR=1·43 (1·33–1·54)  Asthma-related ICU admissions (secondary outcome)  5-11 years: IRR=1·98 (1·03–3·79)  12-17 years: IRR=2·09 (0·88–4·98)  18+ years: IRR=1·35 (0·96–1·89) |

### **Mortality**

| **Outcome** | **Paper** | **Cases (n)** | **Sample size (N)** | **Effect measure and size(s) (2dp)** |
| --- | --- | --- | --- | --- |
| Mortality | To et al. (2014) | N/A | N/A | Asthma-underlying or specific mortality: RR=1·60 (1·16–2·20)  Asthma-contributing mortality: RR=1.34 (1·10–1·64) |
|  | Gupta et al. (2018) | 14,830 | N/A | 5-44: IRR=0·81 (0·69–0·96)  45-74: IRR=1·37 (1·24–1·52)  75+: IRR=1·30 (1·22–1·39) |
|  | Alsallakh et al. (2021) | 750 | 327,906 | IRR=1·56 (1·39–1·76) |
|  | Mukherjee et al. (2022) | N/A | 2110 | β=1·28 (1·10–1·49) |
|  | Khalid et al. (2024) | 1300 | 423,140 | OR=0·77 (0·51–1·15) |

### **Covariates**

| **Authors** | **Mean age (SD)/Median age (IQR)/Age group (n, %)** | **Gender (n, %)** | **Ethnicity (n, %)** | **Mean BMI (SD)/BMI (n, %)** | **Smoking (n, %)** |
| --- | --- | --- | --- | --- | --- |
| Disano et al. (2010) | N/a | N/a | N/a | N/a | N/a |
| Law et al. (2011) | Average annual-weighted percent  18–24: 2038 (8·3%)  25–44: 6111 (6·6%) 45–64 5910 (7·2%) ≥65: 3193 (6·9%) | Average annual-weighted percent  Male: 5489 (5·3%) Female: 11,763 (8·8%) | Average annual-weighted percent  Non-Hispanic white: 11,444 (7·4%)  Non-Hispanic black: 2898 (7·9%)  Hispanic  Puerto Rican: 653 (13·4%)  Other Hispanic: 1686 (4·1%)  Asian: 395 (4·0%)  Non-Hispanic American Indian/Alaskan Natives: 176 (11·3%) | Average annual-weighted percent  Normal or underweight: 5577 (5·9%) Overweight: 5261 (6·1%)  Obese: 6414 (10·2%) | Average annual-weighted percent  Current smoker: 4335 (8·1%) Former smoker: 4209 (7·8%) Never smoked: 8708 (6·4%) |
| Ungar et al. (2011) | Mean (SD): 7·1 (4·1)  1–4: 156 (32%)  >4 to <10: 203 (41%)  10–18: 131 (27%) | Male: 290 (59%) Female: 200 (41%) | N/a | N/a | N/a |
| Auger et al. (2013) | Median age (IQR): 5·1 (2·4−8·7) | Female: ~216 (35·9%) | White/Caucasian: ~234 (38·9%)  Black/African American: ~317 (52·8%)  Other (includes Hispanic): ~50 (8·3%) | N/a | N/a |
| To et al. (2014) | N/a | N/a | N/a | N/a | N/a |
| Zhang et al. (2017) | Median age in months: 141  (~11·75 years old) | Male: ~3172 (57·3%) | White: ~3066 (55·4%)  Black: ~ 952 (17·2%)  Hispanic: ~969 (17·5%)  Not reported: ~548 (9·9%) | Normal: ~3210 (58·0%)  Overweight: ~858 (15·5%)  Obese: ~1467 (26·5%) | N/a |
| Gupta et al. (2018) | Mortality: 5-44: 1370 45-74: 3834 75+: 9067  Admissions: 5-44: 339,580 45-74: 153,946 75+: 49,351 | N/a | N/a | N/a | N/a |
| Mazalovic et al. (2018) | Middle/high SES: ≤25: 71 (31·42%) 26-33: 70 (30·97%) 34+: 85 (37·61%)  Low SES patients: ≤25: 12 (41·38%) 26-33: 7 (24·14%) 34+: 10 (34·48%) | Middle/high SES patients: Male: 101 (44·69%) Female: 125 (55·31%)  Low SES Patients: Male: 8 (27·59%) Female: 21 (72·41%) | N/a | Middle/high SES patients: <25: 120 (63·16%) 25-30: 43 (22·6%) >30: 27 (14·21%)  Low SES patients: <25 = 11 (44·00%) 25-30 = 7 (28·00%) >30 = 7 (28·00%) | Middle/high SES: Non-smoker = 83 (61·03%) Active smoker = 53 (38·97%)  Low SES: Non-smoker: 12(85·71%) Active smoker: 2 (14·29%) |
| Cardet et al. (2018) | Mean age: 40 years  Placebo (n = 193): 39.6 (12·8)  Vitamin D (n = 188):  40.2 (12·5) | Male  Placebo: 62 (32%)  Vitamin D: 61 (32%) | Placebo:  American Indian/Alaskan Native: 0 (0%)  Asian and Pacific Islander: 7 (3.6%)  Black: 64 (33·2%)  White: 103 (53·4%)  Hispanic: 17 (8·8%)  Other: 2 (1·0%)  Vitamin D:  American Indian/Alaskan Native: 1 (0.5%)  Asian and Pacific Islander: 6 (3.2%)  Black: 59 (31·4%)  White: 100 (53·2%)  Hispanic: 19 (10·1%)  Other: 3 (1·6%) | Placebo:  31·6 (9·5)  Vitamin D: 32·3 (8·3) | N/a |
| Grunwell et al. (2018) | Median age (IQR):  Never PICU: 10 (8-13)  Yes (never intubated): 12 (9-14)  Yes (intubated): 12 (9-14) | Female:  Never PICU: 177 (43·3%)  Yes (never intubated): 38 (43·7%)  Yes (intubated): 27 (32·5%)  Male:  Never PICU: 232 (56·7%)  Yes (never intubated): 49 (56·3%)  Yes (intubated): 56 (67·5%) | Hispanic:  Never PICU: 26 (6·4%)  Yes (never intubated): 10 (11·5%)  Yes (intubated): 4 (4·8%)  Non-Hispanic:  Never PICU: 383 (93·6%)  Yes (never intubated): 77 (88·5%)  Yes (intubated): 79 (95·2%) | Normal:  Never PICU: 220 (54·5%)  Yes (never intubated): 40 (45·6%)  Yes (intubated) 39 (47·6%)  Overweight:  Never PICU: 81 (20·1%)  Yes (never intubated): 18 (20·7%)  Yes (intubated): 18 (22·0%)  Obese:  Never PICU: 103 (22·5%)  Yes (never intubated): 29 (33·3%)  Yes (intubated): 25 (30·5%) | N/a |
| Eum et al. (2019) | 2011:  5-9: 514 10-14: 348 15-17: 208  2015:  5-9: 535  10-14: 314  15-17: 213 | 2011:  Male: 565 Female: 505  2015:  Male: 562  Female: 470 | N/a | N/a | N/a |
| Seibert et al. (2019) | Mean age (SD): 30.9 (6·1) | Female 266 (77·8%) | White/non-Hispanic: 48 (14%)  African American/non-Hispanic: 196 (57·3%)  Hispanic: (28·7%) | N/a | Nonsmoker: 237 (69·7%)  Smoker: 103 (30·3%) |
| Brite et al. (2020) | Median age (IQR): 42.0 (35·0-50·0) | Male: 18,585 (61%)  Female: 11,867 (39%) | White: 20,180 (66%)  Black or African American: 3834 (13%)  Hispanic or Latino: 3961 (13%)  Asian, including Native Hawaiian/ Pacific Islander: 1697 (6%)  Multiracial or other: 780 (3%) | N/a | N/a |
| Molina et al. (2020) | 2-4: 192 (29%)  5-11: 388 (58%) 12+: 84 (13%) | Male: 418 (63%) Female: 246 (37%) | African American: 467 (70%)  Other: 197 (30%) | N/a | N/a |
| Jroundi & Tse (2021) | Mean age (SD): 6.5 (4·1) | Female: 26,400 (39·5%) | Black: 25,223 (37·7%)  Hispanic: 15,979 (23·9%)  White: 16,654 (24·9%)  Other: 7491 (11·2%) | N/a | N/a |
| Alsallakh et al. (2021) | Mean age (SD):  47.5 (20·3) | Female (%): (56·3%) | N/a | N/a | N/a |
| Busby et al. (2021) | Mean age (SD):  IMD 1: 50·0 (16·9)  IMD 2: 50·8 (17·0)  IMD 3: 52·1 (17·1)  IMD 4: 51·1 (17·0)  IMD 5: 51·8 (16·7)  *Age groups also reported* | Male:  IMD 1: 6573 (39·8%)  IMD 2: 12,442 (40·1%)  IMD 3: 9944 (40·9%)  IMD 4: 11,120 (41·3%)  IMD 5: 11,853 (42·0%)  Female:  IMD 1: 9961 (60·2%)  IMD 2: 18,617 (59·9%)  IMD 3: 14,388 (59·1%)  IMD 4: 15,780 (58·7%)  IMD 5: 16,362 (58·0%) | IMD 1:  White: 9737 (88·3%) Asian: 933 (8·5%) Black: 185 (1·7%) Mixed: 55 (0·5%) Other: 115 (1·0%)  IMD 2:  White: 20,517 (95·3%) Asian: 769 (3·6%) Black: 143 (0·7%) Mixed: 57 (0·3%) Other: 47 (0·2%)  IMD 3:  White: 15,777 (96·2%) Asian: 449 (2·7%) Black: 61 (0·4%) Mixed: 59 (0·4%) Other: 50 (0·3%)  IMD 4:  White: 16,960 (94·7%) Asian: 710 (4·0%) Black: 104 (0·6%) Mixed: 78 (0·4%) Other: 66 (0·4%)  IMD 5:  White: 18,268 (97·5%) Asian: 304 (1·6%) Black: 45 (0·2%) Mixed: 48 (0.3%) Other: 79 (0.4%) | Mean BMI (SD):  IMD 1: 28·9 (6·4)  IMD 2: 28·6 (6·1)  IMD 3: 28·2 (6·1)  IMD 4: 27·9 (5·9)  IMD 5: 27·8 (5·8) | IMD 1:  Never-smoker: 7929 (50·8%) Ex-smoker: 4206 (26·9%) Current smoker: 3473 (22·3%)  IMD 2:  Never-smoker: 16,135 (53·1%) Ex-smoker: 8744 (28·8%) Current smoker: 5511 (18·1%)  IMD 3:  Never-smoker: 12,492 (55·0%) Ex-smoker: 6549 (28·9%) Current smoker: 3657 (16·1%)  IMD 4:  Never-smoker: 14,977 (57·3%) Ex-smoker: 7483 (28·6%) Current smoker: 3690 (14·1%)  IMD 5:  Never-smoker: 16,376 (59·2%) Ex-smoker: 7672 (27·7%) Current smoker: 3633 (13·1%) |
| Cardet et al. (2022) | Mean age (SD):  49 (±13) | Female: 159 (83·9%) | African American/Black: 504 (50·9%)  Hispanic/Latinx adults: 486 (49·1%) | 25-29.9: 207 (20·9%)  >30: 689 (69·6%) | Current Smoker: 75 (7·6%) Former smoker: 239 (24·1%) Nonsmoker: 676 (68·3%) |
| Mukherjee et al. (2022) | 0-4: (51·1%)  5-9: (22·8%)  10-14: (26·0%) | Male (%): (59%) | N/a | N/a | N/a |
| Kallis et al. (2023) | Age (median, IQR):  Without outcome: 46 ( 33–62)  With outcome: 53 (39–68) | Female (without outcome): 467,576 (58·1%)  Female (with outcome): 61,036 (65·2%) | Without outcome:  White: 709,916 (88·2%)  Black: 26,562 (3·3%)  South Asian: 49,006 (6·1%)  Mixed: 10,534 (1·3%)  Other: 9120 (1·1%)  With outcome:  White: 83,958 (89·7%)  Black: 2,354 (2·5%)  South Asian: 5,543 (5·9%)  Mixed: 961 (1·0%)  Other: 809 (0·9%) | N/a | Without outcome:  Never smoked: 228,308 (28·4%)  Ex-smoker: 362,203 (45%)  Current smoker: 214,627 (26·7%)  With outcome:  Never smoked: 20,720 (22·1%)  Ex-smoker: 43,950 (46·9%)  Current smoker: 28,955 (30·9%) |
| Renzi-Lomholt et al. (2024) | Median age (IQR):  8·0 (4·0-13·0) | Male: 17,646 (59%) | N/a | N/a | N/a |
| Khalaf et al. (2024) | Mean age (SD)  9·7 (3·4) | Male: 65,886 (55·1%)  Female: 53,725 (44·9%) | N/a | Normal: 52,668 (44·1%)  Overweight: 11,367 (9·5%)  Obese: 7575 (6·3%)  Missing: 48,001 (40·1%) | Smoking (Yes): 4225 (3·5%)  Passive smoking (Yes): 2503 (2·1%) |
| Simms-Williams et al. (2024) | Median age (IQR)  Children: 9·1 (7·4–10·6)  Adolescents: 15·5 (13·5–16·5)  Adults: 41·5 (29·5–56·5) | Male:  Children: 54,559 (60·0%)  Adolescents: 67,825 (59·0%)  Adults: 555,856 (47·1%)  Female:  Children: 36,430 (40·0%)  Adolescents: 47,102 (41·0%)  Adults: 623,554 (52·9%) | White:  Children: 55,659 (61·2%)  Adolescents: 56,275 (49·0%)  Adults: 780,056 (66·1%)  Black:  Children: 4252 (4·7%)  Adolescents: 4527 (3·9%)  Adults: 29,490 (2·5%)  Mixed:  Children: 3386 (3·7%)  Adolescents: 2905 (2·5%)  Adults: 17,803 (1·5%)  Asian:  Children: 9834 (10·8%)  Adolescents: 9392 (8·2%)  Adults: 68,409 (5·8%)  Other:  Children: 1375 (1·5%)  Adolescents: 1260 (1·1%)  Adults: 11,304 (1·0%)  Missing:  Children: 16,483 (18·1%)  Adolescents: 40,568 (35·3%)  Adults: 272,348 (23·1%) | Underweight:  Children: 5454 (6·0%)  Adolescents: 15,859 (13·8%)  Adults: 34,438 (2·9%)  Normal weight:  Children: 31,496 (34·6%)  Adolescents: 37,788 (32·9%)  Adults: 379,089 (32·1%)  Overweight:  Children: 7151 (7·9%)  Adolescents: 10,889 (9·5%)  Adults: 334,266 (28·3%)  Obese:  Children: 3216 (3·5%) Adolescents: 5604 (4·9%)  Adults: 297,323 (25·2%)  Missing:  Children: 43,672 (48·0%)  Adolescents: 44,787 (39·0%)  Adults: 134,294 (11·4%) | Current smoker:  Adolescents: 11,749 (10·2%) Adults: 324,947 (27·6%)  Former smoker: Adolescents: 11,970 (10·4%) Adults: 439,116 (37·2%)  Never smoked: Adolescents: 60,832 (52·9%)  Adults: 397,038 (33·7%)  Missing:  Adolescents: 30,376 (26·4%)  Adults: 18,309 (1·6%) |
| Akinyemi et al. (2024) | 18–45:  636,623 (38·2%)  45–65: 416,082 (25·0%)  >65: 612,811 (36·8%) | Female: 945,257 (56·8%) | White: 879,693 (53·5%)  Black: 551,068 (33·5%)  Hispanics: 120,233 (7·3%)  Asian/Pacific Islander: 48,953 (3·0%)  Native Americans: 3,133 (0·2%)  Others: 42,671 (2·6%) | Obesity: 376,214 (22·6%) | Current smokers: 312,036 (18·7%) |
| Gaietto et al. (2024) | Cross-sectional  (Visit 1)  Yes: 9·4 (±2·5) No: 10·1 (±2·4)  (Visit 2)  Yes: 14·9 (±3·0)  No: 15·2 (±2·8)  Longitudinal: N/A | Cross-sectional  (Visit 1)  Female (Yes): 35 (43·8%)  Female (No): 54 (41·9%)  (Visit 2)  Female (Yes): 26 (55·3%)  Female (No): 63 (38·9%)  Longitudinal:  Female (Yes): 18 (58·1%)  Female (No): 71 (39.9%) | N/a | Overweight or obesity:  Cross-sectional (Visit 1)  Yes: 38 (50.0%)  No: 57 (50·4%)  (Visit 2)  Yes: 28 (59·6%)  No: 62 (38·3%)  Longitudinal:  Yes: 15 (50·0%)  No: 54 (34·0%) | N/a |
| Scott et al. (2024) | 18–34: 43 (20%)  35–49: 75 (36%)  50–64: 67 (32%)  65+: 26 (12%) | Male: 72 (34%)  Female: 139 (66%) | N/a | Underweight/normal: 48 (24%)  Overweight: 54 (27%)  Obesity: 98 (49%) | N/a |
| Khalid et al. (2024) | Mean age, years, by season:  Winter: 52·1%  Spring: 51.6%  Summer: 50·3%  Fall: 48·9% | Female by season:  Winter: 74·1%  Spring: 73·7%  Summer: 71·6%  Fall: 72·4% | By season:  Winter  White: 46·6%  Black: 29·9%  Hispanic: 16·5%  Asian or Pacific Islander: 2·9%  Native American: 0·7%  Other: 3·5%  Spring  White: 44·6%  Black: 31·9%  Hispanic: 16·3%  Asian or Pacific Islander: 2·8%  Native American: 0·7%  Other: 3·7%  Summer  White: 42·5%  Black: 35·0%  Hispanic: 15·4%  Asian or Pacific Islander: 2·8%  Native American: 0·7%  Other: 3·6%  Fall  White: 43·5%  Black: 33·3%  Hispanic: 16·6%  Asian or Pacific Islander: 2·5%  Native American: 0·6%  Other: 3·5% | N/a | N/a |
| Skeen et al. (2024) | Median age (IQR): 11·8 (10·15-13·83) | Male: 120 (62·2%)  Female: 73 (57·8%) | Hispanic: 112 (58·0%)  Non-Hispanic: 81 (42·0%) | N/a | N/a |
| Xu et al. (2024) | Mean age (SD): 43·6 (17·8) | Female: 64·0% | Asian/Pacific Islander: 10·0%  Black: 12·4%  Hispanic: 38·3%  White: 36·6%  Other/multiple/unknown: 2·7% | Mean (SD): 30·2 (7·4)  Normal weight: 25·6%  Overweight: 30·3%  Obese: 44·1%  Unknown: 0·1% | Current smoker: 5·3%  Former smoker: 20·2%  Never smoked: 74·3%  Unknown: 0·2% |
| Miller et al. (2025) | Decade born:  1990-1999: 1650 (10·4%)  2000-2009: 4421 (27·8%)  2010-2018: 9806 (61·8%) | Female: 8084 (50·9%)  Male: 7793 (49·1%) | Non-Hispanic White: 8416 (53·0%)  Non-Hispanic Black: 2561 (16·1%)  Hispanic White: 1577 (9·9%)  Hispanic Black: 191 (1·2%)  Other: 3132 (19·7%) | N/a | N/a |

## **Appendix 1: Deriving odds ratio from Shor et al (2017)**^30^ **conversion formula**

Risk ratio in terms of odds ratio (OR):

$$RR=\frac{OR}{\left( 1-r \right)+\left( r\times OR \right)}$$

Risk ratio in terms of hazard ratio (HR):

$$RR=\frac{1-e^{HR\times ln(1-r)}}{r}$$

Equating the two formulas:

$$\frac{OR}{\left( 1-r \right)+\left( r\times OR \right)}= \frac{1-e^{HR\times ln(1-r)}}{r}$$

Cross multiplying to eliminate the denominators:

$$OR\times r=(1-e^{HR\times\ln\left( 1-r \right)})\times(\left( 1-r \right)+\left( r\times OR \right))$$

Expanding the right side:

$$OR\times r=(1-e^{HR\times\ln\left( 1-r \right)})\times\left( 1-r \right)+\left( 1-e^{HR\times\ln\left( 1-r \right)} \right)\times\left( r\times OR \right)$$

Isolating OR on one side:

$$OR\times r-\left( 1-e^{HR\times\ln\left( 1-r \right)} \right)\times\left( r \times OR \right)=\left( 1-e^{HR\times\ln\left( 1-r \right)} \right)\times\left( 1-r \right)$$

Factoring OR out of the left side:

$$OR\times\left( r-r\times(1-e^{HR\times\ln\left( 1-r \right)}) \right)=\left( 1-e^{HR\times\ln\left( 1-r \right)} \right)\times\left( 1-r \right)$$

Deriving OR:

$$OR=\frac{\left( 1-e^{HR\times\ln\left( 1-r \right)} \right)\times(1-r)}{r\times e^{HR\times\ln\left( 1-r \right)}}$$

## **References**

1 Ungar W, Paterson J, Gomes T, *et al.* PRS42 EFFECTS OF ASTHMA MANAGEMENT, SOCIOECONOMIC STATUS AND MEDICATION INSURANCE CHARACTERISTICS ON EXACERBATION FREQUENCY IN CHILDREN WITH ASTHMA. *Value in Health* 2010; **13**: A326.

2 Heffernan P, Day A, Wang M, Lam M, Lougheed D. Socioeconomic Deprivation and Emergency Department Use in Ontario, Canada: A Poisson Regression Analysis. *CHEST* 2011; **140**: 227A.

3 Tamulis T, Krupitsky D. Socioeconomic and Environmental Determinants of Hospitalizations for Childhood and Adult Asthma in Hawaii. *Epidemiology* 2011; **22**: S113.

4 Grecian S, Grecian R, Dermott S, Balata H, Ashish A, Aziz I. The relationship between social deprivation and hospital admissions with asthma. *European Respiratory Journal* 2013; **42**. https://erj.ersjournals.com/content/42/Suppl_57/P957 (accessed April 15, 2024).

5 Von Bülow A, Kriegbaum M, Backer V, Porsbjerg C. Poor asthma control is associated with low socio-economic status: Results from a nationwide cross sectional study of Danish patients with asthma. *European Respiratory Journal* 2015; **46**. DOI:10.1183/13993003.congress-2015.PA2024.

6 Slain K, Broberg M, Stormorken A, Shein S, Rotta A. 518: POVERTY IS ASSOCIATED WITH LONGER HOSPITAL LENGTH OF STAY FOR CHILDREN WITH CRITICAL ASTHMA. *Critical Care Medicine* 2016; **44**: 206.

7 Yakubovich AR, Cluver LD, Gie R. OP29 Are socioeconomic conditions associated with asthma prevalence, severity, and treatment access? A cross-sectional study of children living in low-income South African communities. *J Epidemiol Community Health* 2016; **70**: A20–1.

8 Al Sallakh MA, Rodgers SE, Lyons RA, Sheikh A, Davies GA. Socioeconomic deprivation and inequalities in asthma care in Wales. *The Lancet* 2017; **390**: S19.

9 Chang K-L, Cardet J, Hernandez PA, *et al.* SOCIOECONOMIC STATUS AFFECTS ASTHMA MORBIDITY MEASURES AMONG BLACK AND LATINX PATIENTS. *CHEST* 2021; **160**: A31–3.

10 Håkansson KEJ, Guerrero SC, Backer V, Ulrik CS, Rastogi D. Parental socioeconomic status, access to specialist care, and childhood asthma severity, control, and exacerbations. *European Respiratory Journal* 2022; **60**. DOI:10.1183/13993003.congress-2022.4384.

11 Khamooshi P, Haghani Rad N, Reyes Pinzon V, Yoonsun Kang C, Shaka H. A Decade of Economic Disparity Among Adult Asthma Readmissions in the United States. 2022; published online May. DOI:10.1164/ajrccm-conference.2022.205.1_MeetingAbstracts.A5114.

12 Skeen E, Moore C m., Liu A h., Seibold M a., Hamlington K l. Neighborhood-level Child Opportunity Predicts Exacerbation-prone Status in a Cohort of Urban Children With Asthma. 2023; published online May. DOI:10.1164/ajrccm-conference.2023.207.1_MeetingAbstracts.A4227.

13 Hussain H, McKeever TM, Gonem S. P45 Asthma outcomes, inhaled corticosteroid adherence and socioeconomic deprivation in English clinical commissioning group regions. *Thorax* 2023; **78**: A132–3.

14 Francis-Morel G, Guevara N, Perez E, Aviles DS. ASTHMA AND INCOME: A NATIONAL RETROSPECTIVE COHORT. *CHEST* 2023; **164**: A5007.

15 Padasak CL, Boring S, Engleson A, Hammers J. Deaths from asthma: a series of cases and their presentations, and the impact on local and global health. *American Journal of Clinical Pathology* 2024; **162**: S5–6.

16 Alsallakh M, Holden KA, Davies G. Inequalities in asthma. In: Inequalities in Respiratory Health (ERS Monograph). Sheffield: European Respiratory Society, 2023: 141–52.

17 Schlichting D, Fadason T, Grant CC, O’Sullivan JM. Childhood asthma in New Zealand: the impact of on-going socioeconomic disadvantage (2010-2019). *N Z Med J* 2021; **134**: 80–95.

18 Crighton EJ, Wilson K, Senècal S. The relationship between socio-economic and geographic factors and asthma among Canada’s Aboriginal populations. *International Journal of Circumpolar Health* 2010; **69**: 138–50.

19 Vowles M, Kerry R, Ingram B, Mason L. Investigation of the Environmental and Socio-Economic Characteristics of Counties with a High Asthma Burden to Focus Asthma Action in Utah. *International Journal of Environmental Research and Public Health* 2020; **17**: 5251.

20 Moncrief T, Beck AF, Simmons JM, Huang B, Kahn RS. Single parent households and increased child asthma morbidity. *J Asthma* 2014; **51**: 260–6.

21 Sakai-Bizmark R, Chang R-KR, Mena LA, Webber EJ, Marr EH, Kwong KY. Asthma Hospitalizations Among Homeless Children in New York State. *Pediatrics* 2019; **144**: e20182769.

22 De La Cruz N, Hines JH, Shaw C, *et al.* Geographic Disparity in Asthma Hospitalizations: The Role of Race/Ethnicity, Socioeconomic Status, and Other Factors. *Cureus* 2021; **13**. DOI:10.7759/cureus.20015.

23 Grant TL, Lavigne LCR, Pollack CE, *et al.* Moving to lower-poverty neighborhoods offers broad benefits for children with asthma, regardless of sex or other baseline characteristics. *Journal of Allergy and Clinical Immunology: Global* 2025; **4**. DOI:10.1016/j.jacig.2025.100402.

24 Gaffney A, McCormick D, Bor D, Himmelstein DU, Woolhandler S. Age of Emergence of Disparities in Asthma Prevalence and Morbidity among US Children. *Ann Am Thorac Soc* 2024; published online Oct 15. DOI:10.1513/AnnalsATS.202310-916OC.

25 Lim A, Benjasirisan C, Liu X, *et al.* Social determinants of health and emergency department visits among older adults with multimorbidity: insight from 2010 to 2018 National Health Interview Survey. *BMC Public Health* 2024; **24**: 1153.

26 Thakur N, Martin M, Castellanos E, *et al.* Socioeconomic Status and Asthma Control in African American Youth in SAGE II. *J Asthma* 2014; **51**: 720–8.

27 Kim D, Glazier RH, Zagorski B, Kawachi I, Oreopoulos P. Neighbourhood socioeconomic position and risks of major chronic diseases and all-cause mortality: a quasi-experimental study. *BMJ Open* 2018; **8**: e018793.

28 Antunes FP, Costa M da CN, Paim JS, *et al.* Desigualdades sociais na distribuição espacial das hospitalizações por doenças respiratórias. *Cad Saúde Pública* 2013; **29**: 1346–56.

29 Cardet JC, King TS, Louisias M, *et al.* Income Is an Independent Risk Factor for Worse Asthma Outcomes. *Journal of Allergy and Clinical Immunology* 2016; **137**: AB9.

30 Shor E, Roelfs D, Vang ZM. The “Hispanic mortality paradox” revisited: Meta-analysis and meta-regression of life-course differentials in Latin American and Caribbean immigrants’ mortality. *Social Science & Medicine* 2017; **186**: 20–33.
